# Health Communication and Adherence to Noninvasive Ventilation in Chronic Hypercapnic Respiratory Failure: A Randomized Clinical Trial

**DOI:** 10.1001/jamanetworkopen.2024.51614

**Published:** 2024-12-26

**Authors:** Doris Sau Fung Yu, Polly Wai-Chi Li, Jason Chi-Chun Lau, Pik Shan Alice Cheung, Mary Ip, Suet Lai Linda Cheng, Henry Chung Leung Poon, Fung Kam Iris Lee

**Affiliations:** 1 School of Nursing, Li Ka Shing Faculty of Medicine, The University of Hong Kong, Hong Kong, China; 2 Department of Medicine and Geriatrics, United Christian Hospital, Hong Kong, China; 3 Respiratory and Critical Care Medicine, LKS Faculty of Medicine, The University of Hong Kong, China; 4 Department of Medicine and Geriatrics, United Christian Hospital, Hong Kong, China; 5 Nethersole Institute of Continuing Holistic Health Education (NICHE), Alice Ho Miu Ling Nethersole Charity Foundation, Hong Kong, China

## Abstract

**Importance:**

Domiciliary noninvasive ventilation (NIV) is a standard treatment for improving health outcomes among patients with chronic hypercapnic respiratory failure (CHRF). However, poor adherence substantially limits its therapeutic effectiveness.

**Objective:**

To determine the effect of an information-motivation-behavioral (IMB) skills-based intervention (IMB-NIV program) on NIV adherence, patient-reported health outcomes, and health service use among patients with CHRF.

**Design, Setting, and Participants:**

This multisite, assessor-blinded, randomized clinical trial was conducted from January 2022 to March 2023 at respiratory clinics of 2 regional hospitals in Hong Kong among patients with CHRF who had been nonadherent to prescribed domiciliary NIV.

**Interventions:**

The 6-week IMB-NIV program used a hybrid approach, combining in-person and telecare sessions, to provide patients with knowledge, skills, a positive attitude, and social motivation to enhance NIV adherence.

**Main Outcomes and Measures:**

The primary outcome was NIV adherence data retrieved from the NIV machine. Secondary outcomes included the Pittsburgh Sleep Quality Index, Chinese Severe Respiratory Insufficiency questionnaire, venous bicarbonate (HCO_3_^−^) level, emergency department (ED) and hospital admissions, and mortality measured at baseline, program completion, and 3, 6, and 12 months.

**Results:**

Of the 124 participants (mean [SD] age, 73.7 [7.2] years; 67 [54.0%] female), the 62 patients in the IMB-NIV group were more likely to adhere to NIV compared with the 62 patients in the usual care group at 12 months (61.3% vs 27.4%; odds ratio, 2.78; 95% CI, 1.69 to 4.55), with a greater increase noted in daily NIV use in hours at that time (B = 2.37; 95% CI, 1.44 to 3.31). A generalized estimating equation indicated significantly greater improvements in sleep quality (B = −3.63; 95% CI, −5.14 to −2.12) and health-related quality of life (β = 14.84; 95% CI, 9.18 to 20.49) at 12 months in the IMB-NIV group. The IMB-NIV group had significantly lower ED admissions (incidence rate ratio, 0.47; 95% CI, 0.26 to 0.84) and a longer time to the first ED visit (hazard ratio, 0.51; 95% CI, 0.28 to 0.95). No significant treatment effect was observed on venous HCO_3_^−^ levels or hospital admissions.

**Conclusions and Relevance:**

In this randomized clinical trial, the IMB-NIV program offered a promising behavioral approach to improve NIV adherence in patients with CHRF. Its sustained positive effects on sleep quality, health-related quality of life, and ED admissions highlight its potential to optimize the therapeutic benefits of NIV.

**Trial Registration:**

ClinicalTrials.gov Identifier: NCT05008211.

## Key Points

**Questions:**

Can a motivational behavioral intervention improve adherence to use of noninvasive ventilation (NIV) among adults with chronic hypercapnic respiratory failure (CHRF)?

**Findings:**

This randomized clinical trial with 124 patients found that the information-motivation-behavioral program (IMB-NIV) significantly improved NIV adherence, sleep quality, and CHRF-specific quality of life and reduced emergency department utilization in patients with CHRF, with effects sustained up to the 12-month follow-up.

**Meaning:**

The findings of this study suggest that the IMB-NIV program is a highly feasible, person-centered care model that optimizes NIV use and enhances therapeutic outcomes, including patient-reported health outcomes and reduced health service utilization.

## Teaser

This randomized clinical trial evaluates the effects of an information-motivation-behavioral intervention designed to improve adherence to domiciliary noninvasive ventilation among patients with chronic hypercapnic respiratory failure.

## Introduction

Chronic hypercapnic respiratory failure (CHRF) is a complication arising from various pulmonary, cardiovascular, and neuromuscular disorders.^1^ CHRF is characterized by the accumulation of bicarbonate (HCO_3_^−^) in the venous system. Dyspnea is the hallmark symptom of CHRF, and it substantially impairs sleep quality, vitality, physical function, and psychosocial well-being.^1^ Given that the prevalence of CHRF increases with age^2^ and CHRF remains a frequent cause of hospital admissions,^3^ the associated disease burden is expected to considerably increase in the coming decade due to population aging.

Domiciliary noninvasive ventilation (NIV) is a guideline-recommended management strategy for CHRF that delivers positive airway pressure through a facial mask to improve inspiration. Evidence supports its effectiveness in reducing hospital admissions and mortality.^4^ Other studies have demonstrated the positive effects of NIV on disease-specific health-related quality of life (HRQoL).^5,6^ However, NIV nonadherence remains a major problem due to discomfort or injuries from the mask interface, technical difficulties, air leakage, emotional distress, asynchronous breathing, fear of suffocation, treatment dependence, and social unacceptance.^7,8^ Previous studies have consistently indicated that NIV nonadherence reduces therapeutic benefits related to symptom control, HRQoL, and even mortality.^9,10^ Maintaining an adherence threshold of at least 4 hours per day has been proven to improve respiratory function, gas exchange, and symptom control.^11,12^

Research on improving NIV adherence is limited. To our knowledge, only one trial^13^ has explored the use of cognitive-behavioral interventions to improve NIV adherence and health outcomes. However, this trial relied on self-reported adherence, which may have been affected by social desirability and recall bias.^13^ In addition, the need for frequent sessions with a clinical psychologist limited the intervention’s feasibility, as indicated by the high attrition rate.^13^ Another shortcoming was the absence of strategies to promote long-term motivation, which is critical for sustained NIV adherence.

The information-motivation-behavioral (IMB) skills model provides a framework for addressing complex factors contributing to poor NIV adherence.^14^ In addition to meeting the substantial informational needs of patients with CHRF to support health behaviors, the model can enhance both personal and social motivation. Personal motivation involves cultivating a positive attitude toward NIV use, whereas social motivation focuses on improving patients’ perceived social support and social acceptance in adhering to treatment. Furthermore, the IMB model incorporates a behavioral component that extends beyond treatment-related skill development to increase self-efficacy and perceived control in changing health behaviors. This focus is particularly relevant for addressing maladaptive emotional responses to NIV use. The objective of this randomized clinical trial was to examine the effects of an intervention based on the IMB skills model (ie, the IMB-NIV program) on NIV adherence (primary outcome), serum bicarbonate (HCO_3_^−^) levels, patient-reported health outcomes (sleep quality and HRQoL), and health service use (emergency department [ED] and hospital admissions).

## Methods

### Study Design

This 2-group, multisite, assessor-blinded, randomized clinical trial was conducted in the respiratory clinics of 2 regional hospitals in Hong Kong. The study protocol was approved by the Clinical Research Ethics Committee and adhered to the Declaration of Helsinki. All participants provided written informed consent. The trial followed the Consolidated Standards of Reporting Trials (CONSORT) reporting guideline.^15^ The trial protocol is available in Supplement 1.

### Participants

A research assistant identified potential participants by screening the electronic health records of all patients who attended the respiratory clinics between January 2022 and March 2023. Patients were eligible if they were diagnosed with CHRF, had a partial pressure of carbon dioxide, arterial (Paco_2_), level of 7 kPa (52.5 mm Hg) or greater for at least 4 weeks, had been prescribed domiciliary NIV for 4 or more weeks, and were classified as nonadherent to domiciliary NIV (defined as using NIV for <4 h/night for >70% of days^16^ in the last 2 weeks). Eligible patients also needed to provide informed consent to participate. Patients with known psychiatric disorders (except anxiety and depression), diseases with a life expectancy of 1 year or shorter, and active malignant neoplasms were excluded.

The effect size was estimated based on our pilot study findings. The pilot study indicated that the IMB-NIV program could improve treatment adherence by nearly 40 percentage points (IMB-NIV vs usual care: 70.6% vs 31.6%) at the 12-month end point.^17^ For this trial, we conservatively assumed a between-group difference of 25 percentage points in treatment adherence, with an improvement of 45 percentage points and 20 percentage points in the IMB-NIV and usual care groups, respectively. Power analysis performed using Pass version 14.0 (NCSS) indicated that 62 participants per group would be required to achieve 80% power at a 2-sided 95% CI, accounting for 15% attrition. Participants were randomly assigned to receive either the IMB-NIV program or usual care in a 1:1 ratio through permuted block randomization using block sizes of 4, 6, and 8 with computer-generated sequences. The research assistant used sequentially numbered, opaque, sealed envelopes to ensure allocation concealment.

### Interventions

#### IMB-NIV program

The 6-week IMB-NIV program used a hybrid approach, incorporating home visits, telecare, and clinic visits to improve accessibility for patients with CHRF who are more likely to have decreased activity tolerance. The program was delivered by 3 registered nurses who had strong experience in health counseling. They had received intensive training on using the IMB approach to improve NIV and advanced respiratory care. The training was delivered by advanced practice nurses in respiratory and medical care and nursing academicians who have professional portfolios in using behavioral therapies and motivational interviewing to empower and motivate self-care for chronic disease management. The content covered disease-related knowledge, NIV care and self-care, the IMB model constructs, the role of personal and social motivations in shaping health behaviors, goal-oriented empowerment and motivational strategies, and patient engagement in home visit and telecare. A standardized intervention protocol and toolkit (including information package, goal-setting portfolio, and telephone follow-up documents) were used to guide the practice. For each patient, the same nurse delivered the whole IMB model to ensure continuity of care. They were also supported by a multidisciplinary team, including geriatricians and advanced practice nurses, for program development and implementation. The core of the IMB-NIV program was a person-centered approach aimed at enhancing knowledge and skills for managing CHRF while cultivating a positive attitude and social motivation to promote long-term adherence to domiciliary NIV.^18^ For further details, please refer to the study protocol (Supplement 1).

The program began with a 60-minute home visit during which the nurse assessed the participants’ self-care needs related to CHRF and NIV management. Health education was provided to increase the participants’ awareness of their nonadherent behaviors as well as the underlying misbeliefs and attitudes toward self-care. The potential detrimental health consequences of nonadherence were highlighted to motivate the participants to set self-directed goals and develop an action plan for improving NIV adherence. The goal categories set for the patients are listed in eTable 1 in Supplement 2. A nurse-patient partnership approach was used to address the participants’ concerns, confront negative thoughts, and identify personal and social support resources to facilitate behavioral changes. Skill training focused on proper NIV handling and complication prevention, with an emphasis on optimizing self-care in the home environment. The visit concluded with brainstorming to anticipate barriers to goal attainment and explore potential solutions. If available, family members were involved to provide support, and a telephone consultation hotline, available during office hours, was provided.

Two 20-mintue televisits were scheduled in the second and fourth weeks to monitor progress and review goals. Health counseling was provided to address barriers to NIV adherence and any evolving self-care challenges. The program concluded with a 30-minute follow-up clinic visit aimed at reinforcing long-term behavioral changes that support NIV adherence. Health communication during this visit encouraged participants to reflect on improvements in their NIV self-care and the resulting perceived health benefits. Further behavioral skill training, positive reinforcement of the prognostic benefits of optimal NIV adherence, and practical strategies for integrating self-care into daily routines were provided.

#### Usual Care

Usual care consisted of regular medical follow-ups at the respiratory clinic where a nursing team provided support for troubleshooting problems related to domiciliary NIV use and delivered relevant health education as needed. The IMB group also received this standard care during their medical follow-ups. Although the use of attention-placebo allows a more stringent evaluation of the active components of the tested intervention, it was challenging to ensure its similar dose as the IMB-NIV program (ie, approximately 2-hour interaction in 4 nurse-patient encounters), with the content perceived as credible by the patients but not related to the self-care and quality-of-life outcomes.^19^ Given the education-supportive component of the usual care further limited the content design of the attention placebo, usual care was adopted as the control condition.

### Outcome Measures

All outcomes were assessed at baseline, at IMB-NIV program completion (ie, program exit at the seventh week), and at 3, 6, and 12 months. Data were collected by a nurse blinded to the randomization, either in the clinic or via phone call. For the primary outcome, adherence data, including daily NIV usage over the past 2 weeks, were retrieved from the NIV machine. Adherence was defined as more than 4 hours of nighttime use for more than 70% of days^16^ or a mean daily use of more than 5 hours.

Secondary outcomes included sleep quality, measured using the Chinese Pittsburgh Sleep Quality Index (CPSQI),^20^ and HRQoL, measured using the Chinese Severe Respiratory Insufficiency Questionnaire (CSRI).^21^ Both scales include subdomain scores to assess specific aspects of sleep function and the impact of CHRF on physical and psychosocial well-being. Venus HCO_3_^−^ levels were measured at baseline and at 3 and 6 months. Data on respiratory-related ED admissions and hospital admissions were retrieved from electronic health records over the 12-month period.

### Statistical Analysis

A generalized estimating equation (GEE) model was used to examine the effects of the IMB-NIV program on daily NIV usage, CPSQI scores, CSRI scores, and serum HCO_3_^−^ levels following the intention-to-treat principle. The model was adjusted for potential confounders, specifically baseline clinical and demographic characteristics with a 2-sided *P* < .25.^22^ The time × group interaction term in the GEE model was included to compare mean changes in primary and secondary outcomes between the groups from baseline to the various time points across the 12-month period, and effect sizes (Cohen *d*) were calculated. The relative ratios of becoming NIV-adherent (ie, >4 hours of nighttime use for >70% of days over the past 4 weeks) were computed for each time point and compared between the groups. Negative binomial regression was used to determine between-group differences in ER visits and hospital admissions because these data were likely over-dispersed in the sample. Cox proportional hazards regression analysis was performed to assess between-group differences in time-to-event outcomes, and corresponding hazard ratios were calculated. Statistical analyses were performed using SPSS software version 29.0 (IBM Corp), with 2-sided α = .05 as the threshold for statistical significance.

## Results

### Study Participants

**Figure 1.**
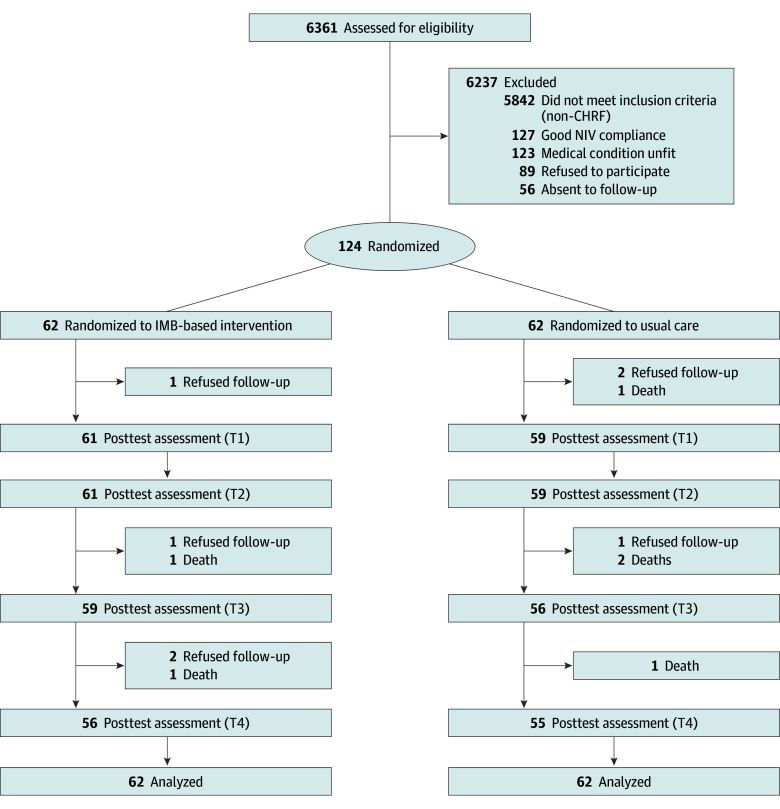
Study Flowchart CHRF indicates chronic hypercapnic respiratory failure; IMB, information-motivation-behavioral; and NIV, noninvasive ventilation.

Of 6361 patients who consecutively attended the respiratory clinics at 2 regional hospitals, 124 patients (mean [SD] age, 70.1 [8.0] years; 67 [54.0%] female) were recruited, with 62 randomized to the IMB-NIV group and 62 to the usual care group (Table 1). Among them, 31 (25.0%) and 64 (51.6%) patients had chronic obstructive airway disease and sleep apnea, respectively. The mean Charlson Comorbidity Index was greater than 4.0 for both groups, indicating a high level of chronic disease burden. Approximately 30% of participants had been hospitalized at least once in the past year. The 2 study groups differed in age, smoking habits, and comorbidity burden; these variables were controlled for in the outcome evaluation. A total of 111 patients completed the 12-month follow-up assessment, with completion rates of 90.3% (56 of 62) and 88.7% (55 of 62) in the IMB-NIV and usual care groups, respectively (Figure 1). No adverse effects related to the IBM-NIV program were reported.

**Table 1.** Baseline Characteristics of the 124 Study Participants

Characteristics	Patients, No. (%)	
	IMB-based intervention (n = 62)	Usual care (n = 62)
Sociodemographic characteristics		
Age, mean (SD), y	70.05 (11.20)	67.34 (14.23)
Sex		
Male	27 (43.5)	30 (48.4)
Female	35 (56.5)	32 (51.6)
Education level		
<Primary	13 (21.0)	12 (19.4)
Primary	20 (32.3)	20 (32.3)
Secondary	28 (45.2)	28 (45.2)
≥University	1 (1.6)	2 (3.2)
Marital status		
Single	10 (16.1)	6 (9.7)
Married	39 (62.9)	46 (74.2)
Divorced	5 (8.1)	6 (9.7)
Widowed	8 (12.9)	4 (6.5)
Living status		
Single	12 (19.4)	9 (14.5)
Spouse only	10 (16.1)	16 (25.8)
Family	39 (62.9)	35 (56.5)
Friend	0	1 (1.6)
OAHR	1 (1.6)	1(1.6)
Smoking status		
Smoker	3 (4.8)	6 (9.7)
Ex-smoker	16 (25.8)	9 (14.5)
Oxygen therapy		
Long-term oxygen therapy	11 (17.7)	10 (16.1)
Oxygen therapy (pro re nata)	4 (6.5)	3 (4.8)
Only with NIV oxygen therapy	7 (11.3)	2 (3.2)
Clinical characteristics		
Major respiratory diagnosis		
Chronic obstructive pulmonary disease	17 (27.4)	14 (22.6)
Obstructive sleep apnea	30 (48.4)	34 (54.8)
Obesity hypoventilation syndrome	13 (21.0)	12 (19.4)
Restrictive lung disease	8 (12.9)	7 (11.3)
Neuromuscular disease	0	2 (3.2)
Atelectasis	1 (1.6)	1 (1.6)
Bronchiectasis	2 (3.2)	5 (8.1)
Interstitial lung disease	2 (3.2)	0
Asthma	11 (17.7)	13 (21.0)
No. of unplanned respiratory related hospitalization in the last year		
0	39 (62.9)	48 (77.4)
1	15 (24.2)	10 (16.1)
2	1 (1.6)	2 (3.2)
3	3 (4.8)	1 (1.6)
4	2 (3.2)	0
≥5	2 (3.2)	1 (1.6)
Blood HCO_3_^−^ level, mean (SD), mmol/L	30.26 (4.74)	30.70 (5.66)
Charlson comorbidity index, mean (SD)	4.71 (1.95)	4.29 (1.99)
NIV usage machine data in 14 d		
Total d used, %	66.96 (38.33)	64.56 (38.45)
Total d used with >4 h mean use, %	41.32 (33.17)	44.05 (33.93)
Mean use per d, h	2.95 (1.83)	2.87 (1.83)
Outcome scores, mean (SD)		
Pittsburgh Sleep Quality Index^a^		
Total score	8.45 (3.74)	8.03 (4.40)
Sleep quality	1.50 (0.76)	1.50 (0.80)
Sleep latency	1.71 (0.96)	1.48 (1.11)
Sleep duration	1.56 (1.02)	1.61 (1.06)
Habitual sleep efficiency	1.03 (1.24)	0.92 (1.22)
Sleep disturbance	1.23 (0.49)	1.23 (0.53)
Use of sleep medication	0.37 (0.94)	0.37 (0.91)
Daytime dysfunction	1.05 (0.98)	0.92 (1.00)
Severe Respiratory Insufficiency Questionnaire (Chinese version)^b^		
Respiratory complaints	58.82 (21.24)	61.06 (23.74)
Physical functioning	54.37 (20.98)	60.48 (27.17)
Attendant symptoms and sleep	58.12 (17.90)	58.41 (21.31)
Social relationships	73.25 (15.93)	73.59 (17.48)
Anxiety	69.27 (20.80)	76.37 (23.51)
Psychological well-being	66.44 (19.80)	68.95 (21.10)
Social functioning	60.53 (22.21)	65.88 (22.24)
Summary scale	62.97 (15.51)	66.82 (17.86)

Abbreviations: HCO_3_^−^, bicarbonate; IMB, information-motivation-behavioral; NIV, noninvasive ventilation; OAHR, old-age home resident.

^a^ Range of 0 to 21, with higher scores indicating poorer sleep quality. Subscales ranges from 1 to 7, with higher scores indicating poorer sleeping function.

^b^ Range of 0 to 100, with higher scores indicating better health-related quality of life.

### Primary Outcome

Based on the criterion of nighttime use for more than 4 hours on more than 70% of days, the IMB-NIV group was more likely to adhere to NIV use (32 participants [51.6%] at 7 weeks; 38 [61.3%] at 12 months) compared with the usual care group (10 participants [16.1%] at 7 weeks; 14 [27.4%] at 12 months) at program exit (odds ratio, 3.13; 95% CI, 1.69-5.88). This positive effect of the IMB-NIV program was sustained through the 12-month end point (Table 2). In addition, the IMB-NIV group showed a significantly greater increase in mean daily NIV use than did the usual care group at program exit (group × time effect: B = 2.09; 95% CI, 1.38-2.81; *P* < .001). This positive change was maintained through the 12-month evaluation (Cohen* d* range, 0.724-0.997).

**Table 2.** Effects of the IMB-NIV Program on NIV Adherence, Sleep Quality, and Health-Related Quality of Life

Patient-reported outcome	Mean (SD)		Treatment effect (95% CI)		Time × group interaction effect by GEE	
	IMB-based intervention	Usual care	Change between time points	Cohen *d*	B (95% CI)	*P* value
**NIV use >4 h/d, %**						
Baseline	41.32 (33.17)	44.05 (33.93)	NA	NA	NA	NA
T1	68.90 (35.49)	43.31 (38.80)	26.97 (15.21 to 38.73)	0.826	27.70 (16.09 to 39.30)	<.001
T2	67.22 (37.98)	43.08 (41.69)	26.26 (13.07 to 39.45)	0.720	27.23 (14.30 to 40.16)	<.001
T3	65.93 (40.27)	43.79 (40.00)	26.62 (13.18 to 40.06)	0.722	26.86 (13.87 to 39.85)	<.001
T4	72.46 (35.14)	45.05 (37.51)	30.77 (18.37 to 43.17)	0.923	29.37 (17.33 to 41.40)	<.001
**NIV daily use, h**						
Baseline	2.95 (1.83)	2.87 (1.83)	NA	NA	NA	NA
T1	5.16 (2.59)	2.96 (1.83)	2.02 (1.29 to 2.75)	0.997	2.09 (1.38 to 2.81)	<.001
T2	5.13 (2.93)	3.16 (2.92)	1.85 (0.97 to 2.73)	0.759	1.94 (1.08 to 2.80)	<.001
T3	5.19 (2.99)	3.24 (3.08)	2.03 (1.03 to 3.03)	0.724	2.03 (01.05 to 3.01)	<.001
T4	5.69 (3.03)	3.24 (2.83)	2.45 (1.52 to 3.38)	0.957	2.37 (1.44 to 3.31)	<.001
**NIV use >4 h/d for >70% of d, No. of participants (%)**						
Baseline	0	0	NA	NA	NA	NA
T1	32 (51.6)	10 (16.1)	NA	NA	3.13 (1.69 to 5.88)^a^	<.001
T2	35 (56.5)	18 (29.0)	NA	NA	1.88 (1.20 to 2.94)^a^	.003
T3	36 (58.1)	14 (22.6)	NA	NA	2.50 (1.52 to 4.17)^a^	<.001
T4	38 (61.3)	14 (27.4)	NA	NA	2.78 (1.69 to 4.55)^a^	<.001
**PSQI score** ^b^						
Baseline	8.45 (3.74)	8.03 (4.40)	NA	NA	NA	NA
T1	8.84 (3.69)	9.42 (3.73)	−0.94 (−2.33 to 0.44)	−0.246	−0.96 (−2.32 to 0.40)	.17
T2	7.51 (3.76)	9.58 (4.05)	−2.43 (−3.90 to −0.95)	−0.593	−2.45 (−3.90 to −1.00)	<.001
T3	7.03 (3.42)	10.21 (3.86)	−3.67 (−5.30 to −2.04)	−0.829	−3.66 (−5.23 to −2.09)	<.001
T4	6.23 (3.64)	9.58 (4.08)	−3.87 (−5.45 to −2.29)	−0.920	−3.63 (−5.14 to −2.12)	<.001
**CSRI summary scale** ^c^						
Baseline	62.97 (15.51)	66.82 (17.86)	NA	NA	NA	NA
T1	65.06 (12.98)	60.89 (16.61)	7.61 (2.40 to 12.83)	0.528	7.66 (2.51 to 12.82)	.004
T2	69.60 (12.92)	59.53 (17.53)	13.51 (8.03 to 19.00)	0.891	13.78 (8.38 to 19.17)	<.001
T3	70.37 (13.22)	61.89 (15.39)	12.35 (6.08 to 18.63)	0.725	12.83 (6.83 to 18.82)	<.001
T4	75.16 (12.04)	64.25 (15.42)	14.62 (8.67 to 20.56)	0.925	14.84 (9.18 to 20.49)	<.001
**Blood HCO_3_** ^−^						
Baseline	30.26 (4.74)	30.70 (5.66)	NA	NA	NA	NA
T2	30.28 (4.15)	30.59 (5.33)	−0.20 (−1.57 to 1.16)	−0.059	−0.11 (−1.47 to 1.26)	.87
T3	29.80 (5.00)	30.52 (5.30)	−0.80 (−2.33 to 0.73)	−0.200	−0.49 (−2.00 to 1.02)	.53

Abbreviations: CSRI, Chinese version of the Severe Respiratory Insufficiency Questionnaire; GEE, generalized estimating equation; HCO_3_^−^, bicarbonate; IMB, intention-motivation-behavioral; NA, not applicable; NIV, noninvasive ventilation; PSQI, Pittsburg Sleep Quality Index; T1, 7 weeks after randomization; T2, 3 months after randomization; T3, 6 months after randomization; T4, 12 months after randomization.

^a^ Presented as risk ratio.

^b^ Range of 0 to 21, with higher scores indicating poorer sleep quality; PSQI subscale scores range from 1 to 7, with higher scores indicating poorer sleeping function.,

^c^ Range of 0 to 100, with higher scores indicating better health-related quality of life.

### Secondary Outcomes

The IMB-NIV group demonstrated greater improvements in sleep quality and HRQoL than did the usual care group (Table 2). In particular, the IMB-NIV group showed significantly greater improvements in the overall score and the 7 domain scores of the CPSQI starting at the 3-month follow-up (eTable 2 in Supplement 2). These effects were sustained through the 12-month end point (Cohen *d* range, 0.59-0.92). Similarly, greater improvements in the CSRI summary and domain scores were observed in the IMB-NIV group throughout the evaluation period, from program exit to the 12-month follow-up (Cohen *d* range, 0.53-0.93). However, no significant differences in serum HCO_3_^−^ levels were noted between the study groups.

**Figure 2.**
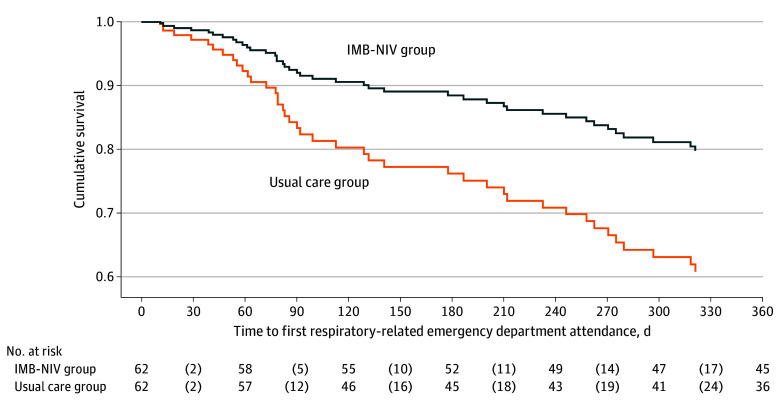
Kaplan-Meier Curves for Time to Emergency Department Attendance Since Randomization After controlling the clinical-demographic variables which were different at *P* < .25, the information-motivation-behavioral noninvasive ventilation (IMB-NIV) group had significantly delayed time to emergency department admission, with a hazard ratio of 0.51 (95% CI, 0.28-0.95).

**Figure 3.**
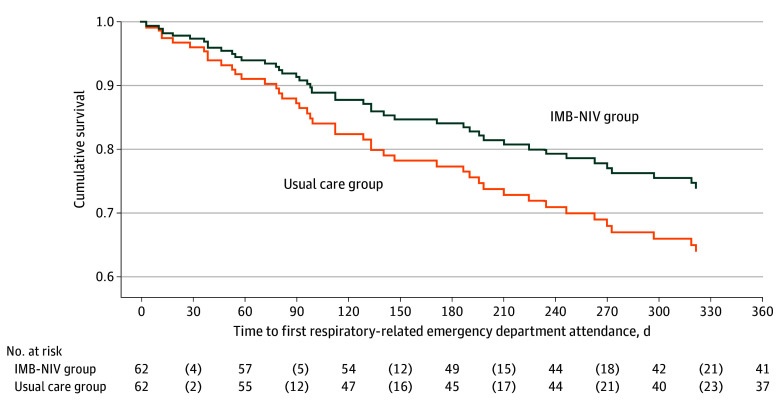
Kaplan-Meier Curves for Time to Hospital Admission Since Randomization After controlling the clinical-demographic variables which were different at *P* < .25, there was no significant between-group difference in the time to hospital admission (hazard ratio, 0.69; 95% CI, 0.38-1.24). IMB-NIV indicates information-motivation-behavioral noninvasive ventilation.

The IMB-NIV program significantly reduced unplanned ED visits during the 12-month evaluation period. Negative binomial regression indicated that the incidence rate ratio (IRR) for ER admissions in the IMB-NIV group (27 admissions) vs the usual care group (66 admissions) was 0.47 (95% CI, 0.26-0.84; *P* = .01). However, there was no difference in hospital admissions between the IMB-NIV and usual care groups (44 vs 65; IRR, 0.69; 95% CI, 0.40-1.19; *P* = .18). Figures 2 and 3 show the survival curves for respiratory-related ED visits and hospital admissions, respectively. Cox proportional hazards regression analysis indicated that the IMB-NIV program significantly delayed the time to respiratory-related ED visits, with a hazard ratio of 0.51 (95% CI, 0.28-0.95) but did not significantly affect time to hospital admissions (hazard ratio, 0.69; 95% CI, 0.38-1.24).

### Subgroup Analysis

As the effects of IMB-NIV program on NIV adherence and hospital services use may vary among patients with different CHRF etiologies (eg, chronic obstructive pulmonary disorder, obstructive sleep apnea alone, obstructive sleep apnea comorbid with other respiratory disorder), subgroup analysis was conducted. The IMB-NIV program significantly improved NIV adherence for all 3 three subgroups (eTables 3-5 in Supplement 2). As for health service use at 12 months, positive intervention effects were observed in the COPD subgroup, who had greater reduction in ED admissions (IRR, 0.23; 95% CI, 0.09-0.62) and hospital admissions (IRR, 0.40; 95% CI, 0.16-0.99). For the other subgroups, no treatment effect on hospital service use was noted.

## Discussion

To our knowledge, this is the first fully powered randomized clinical trial to evaluate the effectiveness of a behavioral strategy aimed at promoting NIV adherence in patients with CHRF. Based on the IMB skills-based model, the IMB-NIV program significantly improved adherence to domiciliary NIV use, and this effect was maintained for up to 12 months. The program also provided extended benefits in sleep quality, symptom control, physical function, and psychosocial adaptation, further reinforcing the therapeutic value of NIV adherence in patients with CHRF. Although no significant improvements were observed in venous HCO_3_^−^ levels or respiratory-related hospital admissions, the reduction in ED visits and the delayed time to these events indicate the program’s additional impact on health service utilization. The hybrid approach, combining in-person visits with telecare, contributed to the program’s full attendance, indicating its feasibility for clinical application.

The IMB model suggests the presence of mutual feedback loops between adherence information, motivation, and actual behavioral changes.^14^ The 6-week program, which included televisits and clinic visits, offered an effective platform to reinforce these 3 core components continuously. This likely explains the sustained improvement in NIV adherence observed up to the 12-month end point. Conversely, a study that used cognitive-behavioral therapy without a motivational component was unable to maintain the initial increase in NIV adherence.^13^ Three major differences in the intervention design compared with our study may explain the variation in the treatment effect.

First, our health communication contrasted patients’ self-reported decision-making processes regarding NIV use with corresponding professional perspectives. This interactive, person-centered approach helped increase patients’ self-awareness of any misconceptions or maladaptive behaviors related to disease management. However, the study using CBT^13^ adopted a more 1-way communication which may only improve the participants’ cognitive understanding of disease and treatment. Second, our study tried to increase intrinsic motivation to change by providing patients with information on the potential detrimental health consequences associated with their maladaptive behaviors. This arrangement created what is called a fear appeal, which was shown to effectively influence attitudes and intentions toward behavioral changes.^23^ In contrast, the CBT study focused on using cognitive restructuring and relaxation to alleviate the anxiety and emotional arousal toward the NIV use. This may enhance extrinsic motivation which is known to be less powerful than intrinsic motivation to secure long-term commitment.^24^ Third, even though both studies taught patients behavioral strategies to increase NIV use, our study initiated a patient-directed goal-setting process to tailor behavioral changes, with particular focus placed on combating the common barriers to NIV adherence (including NIV-induced discomfort, emotional response to asynchronized breathing, fear of dependency, and social rejection). Such an experience of goal attainment would increase one’s sense of self-efficacy and self-control, which further motivate positive behavioral changes.^14,25^ In fact, our findings are consistent with those of a previous study that validated the IMB skills-based model in addressing the complex causes of poor self-care in chronic disease management.^26^

Another factor contributing to the long-term improvement in NIV adherence may be enhanced sleep quality, respiratory symptom control, physical functioning, and psychosocial well-being experienced by participants in the IMB-NIV group. These improvements likely reinforced the health communication provided by the nurse regarding the therapeutic benefits of increased NIV adherence. The perceived health gains may have created a positive feedback loop, motivating sustained behavioral changes. Among the various patient-reported outcomes, sleep quality was not significantly improved until the 3-month end point, indicating that these patients may need time to adjust to sleep disturbances associated with increased NIV use. These findings indicate the need for additional support during the initial stages of behavioral changes to address negative thoughts and provide encouragement. The televisits effectively served this role, helping to sustain adherence behaviors.

Another notable finding is related to the effects of the IMB-NIV program on health service use. The program significantly delayed the time to respiratory-related ED visits and reduced the number of episodes, but it did not affect hospital admissions. This outcome may be attributable to the less developed system of primary care physicians in Hong Kong, where patients with more debilitating CHRF are more likely to seek ED services during disease exacerbation. The results revealed that the control group was more likely to use ED services even when symptom worsening did not warrant hospital admission. This reduction in unnecessary ED visits in the IMB-NIV group may be associated with improved tidal volume and more favorable breathing patterns resulting from increased NIV adherence. NIV delivers positive airway pressure, which increases lung volume, reduces hyperinflation, and enhances the elastic recoil of the chest wall. These effects, in turn, improve carbon dioxide elimination and reduce perceived breathlessness.^27^ Perceived breathlessness was identified as a strong trigger for seeking emergency hospital care.^28^ However, because the higher ED attendance in the usual care group did not lead to a significant difference in hospital admissions between the groups, this increased health service use may not have been necessary to address a severe pathophysiological issue. Thus, the IMB-NIV program likely reduced health service use by improving NIV adherence.

An alternative explanation for the lack of program effect on hospital admission may be related to the mixed CHRF etiologies in our sample. Indeed, the subgroup analysis indicated that the IMB-NIV program significantly reduced both ED admission and hospital admission of those with COPD. This finding aligns with a systematic review, which reported the positive effects of NIV on hypercapnic patients with COPD.^29^ Nevertheless, caution is needed in interpreting the results of the underpowered subgroup analysis for other etiology groups. Future clinical trials on improving NIV adherence need to consider the mixed etiology of the CHRF patients.

The IMB-NIV program did not improve serum HCO_3_^−^ levels. This may be because reductions in venous HCO_3_^−^ levels are more likely to be observed when domiciliary NIV is actively in use.^30,31^ Because blood samples were collected in the clinic, venous HCO_3_^−^ may not have been adequately sensitive to capture the benefits of NIV adherence. Instead, it may be more appropriate to assess ventilatory responses, such as forced expiratory volume in 1 second and total tidal volume, to better reflect these effects.^31^

### Limitations

The study has several limitations. First, the IMB model emphasizes enhancing social motivation by optimizing patients’ social support. However, we did not exclude patients living in singleton or doubleton households, which may have limited the effectiveness of health counseling and threatened the internal validity of our findings. Second, the follow-up period was limited to 12 months, and the small number of patients who died (n = 5) prevented meaningful survival analysis of mortality outcomes. Third, because of the limitations of the NIV machine, we could only retrieve usage data in 2-week intervals. The use of this short reference period necessitates caution when interpreting the study’s findings. Fourth, lack of an attention-placebo as the control may threaten the internal validity of the findings as the effects of the attention from the nurse on the study outcomes cannot be precluded. Fifth, the involvement of a small group of registered nurses to deliver the IMB model also limits the generalizability of the study findings. Additionally, as we did not record the specific time spent with the patients in the intervention arm, its effect cannot be adjusted in the outcome evaluation.

## Conclusions

This study demonstrated the positive effect of a behavioral strategy based on the IMB model in improving NIV adherence in patients with CHRF. The extended benefits of the IMB-NIV program for sleep quality, HRQoL, and ED service use, along with the high fidelity of the hybrid approach, suggest its efficacy to enhance CHRF management. Future studies should evaluate its effects on ventilator parameters and validate its overall clinical effectiveness and cost-effectiveness.

## Notes

Supplement 1.
Trial Protocol

Supplement 2. **eTable 1.** Statistics on Goal-Setting for the Intervention Group **eTable 2.** Effects of the Information-Motivation-Behavioral–Noninvasive Ventilation (IMB-NIV) Program on Sleep Quality and Health-Related Quality of Life **eTable 3.** Subgroup Analysis of the Effects of the Information-Motivation-Behavioral–Noninvasive Ventilation (IMB-NIV) Program on NIV Adherence **eTable 4.** Subgroup Analysis of the Effects of the Information-Motivation-Behavioral–Noninvasive Ventilation (IMB-NIV) Program on Time to Hospital Service Use **eTable 5.** Subgroup Analysis of the Effects of the Information-Motivation-Behavioral–Noninvasive Ventilation (IMB-NIV) Program on Number of Emergency Department Admissions and Hospital Admissions

Supplement 3.
Data Sharing Statement
