# Supplementary material for: Health Communication and Adherence to Noninvasive Ventilation in Chronic Hypercapnic Respiratory Failure: A Randomized Clinical Trial
Source: JAMA Netw Open. 2024 Dec 26;7(12):e2451614. doi: 10.1001/jamanetworkopen.2024.51614 (PMC11672160; doi:10.1001/jamanetworkopen.2024.51614)
Supplement: Supplement 1. — Trial Protocol [file jamanetwopen-e2451614-s001.pdf]

Supplementary material 1: Study Protocol

I. BACKGROUND

CHRF is an ongoing respiratory condition characterized by progressive accumulation of bicarbonate (HCO_3^-) in the venous system, feeling of breathlessness is the hallmark symptom of CHRF which greatly compromises one's sleep quality, vitality, physical function and psychosocial well-being.[1] As the CHRF prevalence increases drastically with age,[2] and is a frequent cause of hospital admission,[3] the associated disease burden would be on a high rise in the coming decade of population aging.

Domiciliary non-invasive ventilation is a guideline-based management for CHRF. By directing positive airway pressure via a facial mask to enhance inspiration, substantial evidence has shown its positive effects on reducing hospital admission and mortality.[4] Regarding COPD patients with CHRF, nocturnal domiciliary NIV is prescribed to improve sleep time and efficiency[5], alleviate nocturnal hypoventilation and daytime hypercapnia[6], reduce respiratory muscle fatigue[7] and improve respiratory mechanics[6, 8]. The benefits of domiciliary NIV in chronic hypercapnic COPD patients including improvements in arterial blood gases, health-related quality of life and mortality were evidenced in the results of some recent studies.[9-15] For instance, these benefits were demonstrated in the results of Kohnlein and colleagues' prospective multi-center RCT[12] when moderate intensity NIV was used and patients with borderline hypercapnia or low adherence were excluded. Domiciliary NIV was also shown to prolong the time to readmission or death in patients with persistent hypercapnia after a COPD exacerbation, when compared to oxygen alone.[16]

Adherence is an important factor affecting the clinical effectiveness of domiciliary NIV. Previous studies indicated that NIV non-adherence posed major challenges to compromise the therapeutic benefits on symptom control, HRQoL and even mortality,[17-18] with an adherence threshold defined as at least 4 hours/ day against improved respiratory function, gaseous exchange and symptom control.[19-20] Lower percentage of days of domiciliary NIV use ≤ 4 hours/day was correlated with increased hospitalization requiring acute NIV salvage ($P = 0.042$).[21] Our team has previously identified various factors associated with domiciliary non-adherence including machine noise ($P = 0.001$), difficulty in breathing ($P = 0.004$), conjunctivitis ($P = 0.004$), excessively high air pressure ($P = 0.006$), sleep disturbance ($P = 0.017$) and

headache (P = 0.029).[21] Other studies also identified air leakage, emotional responses along with asynchronized breathing, fear of suffocation and treatment reliance, as well as social acceptance as the hindering factors for domiciliary NIV use.[22-23]

Although adherence is fundamental to the efficacy of domiciliary NIV in patients with CHRF, there are very few studies to improve the NIV adherence in patients with CHRF. There are two qualitative studies[24-25] on chronic hypercapnic patients' adherence to domiciliary NIV. The qualitative findings of these studies indicate that patients or carers are in needs of informational support such as understanding of nocturnal hypoventilation and its consequences[25], technical and self-care support such as guidance on skin protection[25], guidance on ways to avoid dry mouth[24], and psychosocial support such as acknowledging patients' emotional reactions[25], promoting patients' confidence in the benefits of NIV, and supporting carers and family[24-25]. All these care needs for promoting domiciliary NIV adherence also echo with the findings on factors associated with non-adherence behaviors.[21-23] Up to our knowledge, there is only one trial used cognitive-behavioral intervention to improve NIV adherence and the health outcomes.[13/23] However, the use of self-reported adherence was subject to social desirability and recall bias. The reliance on clinical psychologist with intensive meetings may also compromise the fidelity as evidence by the high attrition rate.[13/23] As NIV requires long-term adherence, no intervention component to address the motivational dimension of behavior change may also be a limitation.

Improving treatment adherence is associated with proactive behavioral changes. Behavioral theories provide a good foundation to inform the care conceptualization to enhance treatment adherence. Among these theories, the information-motivation-behavioral (IMB) skills model may provide an especially relevant framework to address the complex determinants of poor NIV adherence.[14] According to Fisher et al.[26], the IMB skills model proposes that information, motivation, and behavioral skills are the major determinants correlated with performance of health-behavior. It also specifies a set of causal relationships among these constructs. The model is presented in Figure 1. According to the model, it is necessary for to provide an individual with comprehensive information to commit the desired health behaviors. Increasing one's personal and social motivation is an essential pre-requisite to enable and sustain the behavioral changes. Whereas personal motivation concerns with cultivating positive attitude towards the NIV use, social motivation is about the ways to optimize their perceived social support and social

acceptance in treatment compliance. As for the behavioral component, the IMB skills model also goes beyond empowering the clients with skills on managing the treatment required adherence but also their self-efficacy and self-control on the behavioral changes. This emphasis would be the most relevant to address the maladaptive emotional responses to NIV usage. In sum, the IMB skills model highlights that the information and motivation constructs interact with each other to directly and indirectly via the behavior skills construct to affect the performance of health-behavior.

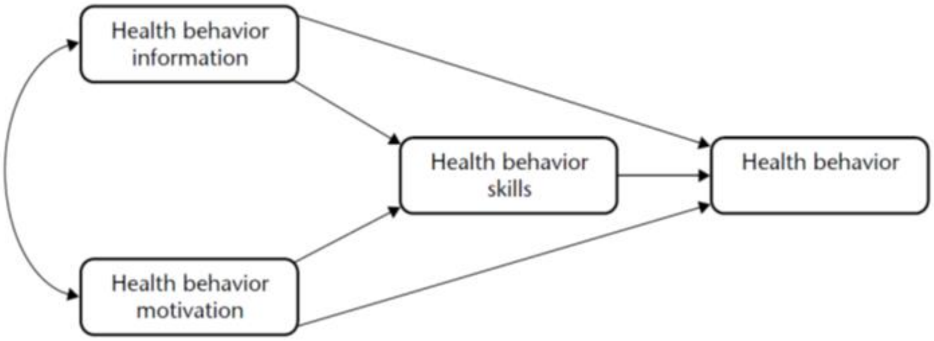

eFigure 1. The Information-Motivation-Behavioral Skills Model of health behavior.[37]

The IMB skills model has been validated in empirical studies or the causal relationships among its constructs and confirmed its predictive validity.[26-28] Earlier studies confirmed its applicability in patients with AIDS,[28] later studies further confirmed its applicability in smoking cessation and patients with chronic health problems including cancer, type 2 diabetes, coronary heart disease, and knee osteoarthritis[28-31]. Regarding domiciliary NIV adherence in patients with CHRF, the previous qualitative findings on the patients' needs of support are well aligned with the information, motivation, and behavioral skill constructs of the IMB model.[24-25] The effects of intervention strategies based on IMB skills model for health behavior change were supported by the results of a systematic review.[28] Varying with duration of intervention, the intervention effects ranged from immediately after to one year post-intervention. Strategies for integrating IMB skills model constructs in intervention included various educational materials and teaching skills for providing information; counseling, motivational strategies and mobilize social support

at family level and beyond for increasing motivation; teaching objective skills and enhancing self-efficacy for developing behavioral skills.[27-31]

Our team conduct the first pilot RCT to develop and evaluate an intervention based on the IMB skills model for promoting patients' adherence to inhalation therapy in COPD.[32] This was the first pilot RCT (N=30) which randomized the participants to receive the intervention based on the IMB skills model or the usual care. The tested intervention consisted of a one-hour face-to-face session for information provision, motivation enhancement and behavioral skill training. A person-centered action plan was developed to optimize the self-care of inhalation therapy. Two telephone follow-up visits were scheduled in the second and fourth weeks, with the purpose to review the action plan implementation and provide informational and psychosocial support for reinforcing adherence. The results supported the effectiveness of this four-week IMB model-based intervention for improving inhalation adherence (ß[95%CI] = 2.01[0.38, 3.63], $P = 0.015$) and inhalation techniques (ß[95%CI] = 15.57[0.385.73, 25.42], $P = 0.002$). Moreover, the intervention was found feasible for application in the local clinical setting and was well accepted by the patients.

The overall aim of this study is to extend the use of the IMB skill-based model to enhance the adherence of domiciliary NIV and the health outcomes including venous bicarbonate (HCO_3^-), patient-reported health outcomes and health service use.

II. RESEARCH AIM & OBJECTIVES

The overall aim of this study is to examine the effect of the IMB-NIV Program which based on information motivation behavioral (IMB) skills model to improve the adherence of domiciliary NIV and the health outcomes of patients with chronic hypercapnic respiratory failure (CHRF). The primary outcome is the NIV adherence, which defined as the use of domiciliary NIV for at least 4 hours per night for at least 70% of the days or a mean daily use of at least 5 hours per day. The secondary outcomes include venous (HCO_3^-), patient-reported health outcomes (including sleep quality, health-related quality of life) and health service use (unplanned hospital admissions related to hypercapnic respiratory failure and survival). All the

outcomes except venous (HCO_3^-) are measured at baseline, post-intervention, and 6th and 12th month. The venous HCO_3^- are assessed up to the second post-test endpoint at 6th month.

.

III. **REASEARCH METHODS**:

1. Study design

It is a multi-site, assessor-blind, randomized controlled trial. The intervention group will receive an IMB-NIV program while the control group will receive the usual care. The study will comply with the Declaration of Helsinki.

2. Subjects

This study will recruit patients with CHRF, who are non-adherers to domiciliary NIV, from the respiratory clinics of two regional hospitals in Hong Kong. The inclusion criteria include: patients who are: (1) diagnosed with CHRF (i.e., $PaCO_2 \geq 7$ kPa of 52.5mmHg) for at least 4 weeks; (2) prescribed with domiciliary NIV for ≥ 4 weeks, and (3) non-adherer (i.e., used domiciliary NIV for < 4 hours per night for > 70% of days or with a mean daily use < 5 hours per day in the last 2 weeks). Patients with known psychiatric disorders except anxiety and depression, disease limiting life expectancy ≥ 1 year, and active malignancy were excluded.

The effect size is estimated from our pilot study that IMB-NIV can improve treatment adherence by almost 40% (IMB-NIV vs usual care: 70.6 vs 31.6) at 12-month endpoint.[33] We conservatively assume an effect size of 25% between-group in treatment adherence with 45% and 20% improvement in the IMB-NIV and usual care group respectively. Power analysis by Pass 14.0 (NCSS, Kaysville, Utah) indicated 62 participants per group is needed to achieve 80% power at 2-sided 95% confidence interval, allowing for 15% attrition. Permutated block randomization using the block size of 4, 6, and 8 and computer-generated sequences for block size and within-block group status assigned the participants to receive the IMB-NIV program or usual care in an 1:1 ratio. A sequence of grouping identifiers (I & C) based on computer-generated random codes will be prepared in advanced by an independent statistician. After obtaining informed consent, group allocation of each participant will be assigned sequentially

according to his/her sequence of enrolment and the corresponding group identifier in the priori prepared random grouping list. The participants will be blinded to their group allocation.

3. Study intervention: The IMB-NIV Program

The 6-week IMB-NIV Program adopted a hybrid approach including 1-hour face-to-face home visit, two telephone follow-ups and one 30-minute clinic visit to increase the accessibility to the CHRF patients who are more likely to have compromised activity tolerance. A telephone consultation hotline during office hour is provided. The program was delivered by a registered nurse who had received training in advanced respiratory care, and a multi-disciplinary team including geriatricians and advanced practice nurses support the program development and implementation.

The Program will be commenced with a home visit during which the nurse assesses the participant's self-care needs (including information, motivation and behavioral skills) on CHRF and NIV. Health education on self-care will be given in a way so that the participants' misconception and ineffective illness behaviors. Information will be provided with assistance of audio-visual materials, as appropriate. The information content includes knowledge of the disease, knowledge of nocturnal hypoventilation and its consequences, the benefits of NIV, how to operate and maintain the ventilator, what are and how to follow the NIV prescription, common problems in using NIV, how to solve the problems, and the concomitant health consequences of poor NIV adherence. This arrangement aims to motivate the followed participant-centered goal settings and action planning to enhance NIV adherence. A nurse-patient partnership approach will be used, together with various strategies for enhancing personal and social motivation towards NIV adherence including establishment of rapport (e.g., acknowledging the patient's experience and emotions towards the disease and use of domiciliary NIV), emphasizing the benefits of domiciliary NIV, involving family to support the patient, individual counseling (e.g., encouraging the patient to express feelings, concerns and problems encountered, clarifying misbeliefs and confront negative thoughts, working with the patient to identify possible solutions, respecting and supporting the patient's choices in problem-solving), making agreement on a goal-oriented action plan, providing positive reinforcement (e.g., review and praise for

achievements and mastery of skills in NIV adherence) and psychosocial support (e.g., active listening and therapeutic communication).

Behavioral skill training on proper NIV handling and complication prevention will be provided with emphasis on technical skills for operating the ventilator at home environment, handling interface (e.g., reducing air leak) and ventilator maintenance, and self-care skills (e.g., skin and eye protection). Strategies will include verbal instructions, physical demonstration, return-demonstration, constructive feedback and positive reinforcement in conjunction with assistance of audio-visual materials. The visit will be closed with a brainstorming on anticipated barriers for goal attainment and the corresponding resolving methods are discussed. Family will be involved to support the patient if available and a telephone consultation hotline during office hour is given.

Two tele-visits were scheduled at 2nd and 4th week for goal monitoring and review. Further health counseling on barriers on NIV adherence and newly evolved self-care challenges were given. The program was ended with a follow-up clinic visit, with the aim to substantiate long-term behavioral changes to favor NIV adherence. Health communication was done in a way to support the participants to reflect on the changes in NIV self-care and the concomitant perceived health consequence. Positive reinforcement was given to highlight the prognostic benefits of optimal NIV adherence. Further behavioral skill training will be given as appropriate. Explicit methods to support longer-term motivation and effective self-care maintenance were discussed. The intervention content is presented in eTable 1.

eTable1. Content of the IMB-NIV program

1st week<br>Empowerment cycle<br><br>60-minute home visit	Person-centered assessment to identify patients' self-care practice on NIV management:<br>• Self-care for the CHRF and the respiratory disorders including symptom monitoring management, medication management, activity pacing and sleep-related self-care<br>• The management of domiciliary NIV at home including technical operation of the NIV, strategies for self-monitoring and prevent complications.<br>• Perceived function of NIV<br>• Perceived benefits of NIV<br>• Barriers for NIV adherence and resolving methods

Information giving: [offer an information booklet/ self-care cards]

Providing the patients with structure education on the following topic. The education is tailored to the patient's needs as identified in the above-mentioned assessment.:

- knowledge of the disease and nocturnal hypoventilation,
- awareness on relationship between NIV and prognosis;
- self-care for NIV with skill demonstration and return demonstration
- common problems in using NIV and resolving methods

NB. The information booklet and self-care card provided a more structured template so that the nurse can put down more person-centered information to the template to tailor the self-care information for the patients.

Highlight self-care deficit

Highlight the discrepancy between the optimal NIV care and patients' reported self-care. Based on the discrepancy in self-care, the nurse elaborates the corresponding detrimental health consequences. The purpose of this step is to increase the intrinsic motivation of the patients in making more proactive behaviroal changes in managing the disease and NIV use. This step also increases the patients' readiness and preparedness to set goals to guide the behavioral changes on NIV adherence

Support self-care goal setting and action planning

Formulating short-and medium goals to enhance NIV self-care and adherence & action plan.

[provide log-book to monitor the level of goal attainment]

Resolve prominent concern for goal attainment:

Reviewing the goals and action plan with the patients. The nurse encourages the patients to think of any anticipated challenges and barriers which might affect the action plan implementation. The nurse will work with the patients to identify the potential resources to tackle the barriers, and to brainstorm how to mobilize such resource to support the action plan implementation and thereby the goal attainment. If necessary, the goals and action plan will be adjusted in order to optimize

	its implementation feasibility. The nurse will also discuss any other concern about action plan implementation and provide the patient with resolving methods.<br><br><u>Behavioral skill building</u><br>The nurse will revisit the skills of the patients in using the NIV including fitting the mask, checking for leakage, humidification, preventing and monitoring for skin pressure, positioning, operating the NIV machine, storage and care of the tubing and masks. Demonstration and return demonstration will be provided. Other self-care skills required for goal attainment such as breathing techniques, upper limb strengthening exercise, activity pacing, relaxation-based practice for emotional control will also be provided according to the actions planned for goal attainment.
2nd week<br>20-min telephone intervention.	Evaluate the action plan implementation and the level of goal attainment.<br>Identify any barriers, and use a partnership approach to provide resolution.<br>Provide health education and counseling to support the goal attainment.<br>Adjust the goals if necessary.<br><br>[Nursing documentation: telephone record]
4th week<br><br>20-min telephone intervention.	Monitor the level of goal attainment and action plan implementation<br>Provide further health counseling and advice for the reported barriers and challenges in goal attainment.<br>Goal adjustment as appropriate.<br><br>[Nursing documentation: telephone record]
6th week<br><br>30-mintue clinic visit	30-min face-to-face follow-up session at hospital/ at patient's home<br>- guided reflection on patient-reported change in NIV management over the past few weeks and the success in coping with challenges and barriers;<br>- support the patients to identify associated perceived health-related changes, if any;<br>- discuss challenges and methods to sustain the positive behavioral changes, if any.<br>- discuss any shortcomings in self-care and advise on the resolving methods

4. **Study intervention: Usual Care**

The usual care included regular medical follow-up in the respiratory clinic by a medical consultant. A nursing team would support problem-shooting on using the domiciliary NIV and provided the health education accordingly. The IMB group received the same usual care during the medical follow-up. The participants in the IMB-NIV program will also receive the same usual care during the medical follow-up.

5. Outcome measures

All outcomes were collected at baseline, upon completion of the IMB-NIV (i.e, program exit at the 7th week), and at 3rd, 6th and 12th months. Another nurse without information about the participants' group status will collect the data. The primary outcome is the domiciliary NIV adherence and the secondary outcomes including sleep quality, health-related quality of life, and venous bicarbonate. Following are the outcome evaluation methods.

<u>Domiciliary NIV adherence</u>:

It is the primary outcome that the machine data will be retrieved. The records from the software fitted on the subject's NIV machine for the past two weeks will be reviewed to determine adherence or non-adherence, and assessed for the percentage of days with usage of at least 4 hours per night and the mean of daily use.

<u>Sleep quality</u>

The Chinese Pittsburgh Sleep Quality Index (CPSQI) will be used to assess the subject's sleep quality.[34] The CPSQI is a 19-item self-reported measures for assessing sleep quality over last month. Seven component scores including subjective sleep quality, sleep latency, sleep duration, habitual sleep efficiency, sleep disturbances, sleep medication, and daytime dysfunction can be obtained from the items. The component scores range from 0 (no problem) to 3 (severe problem) and an overall score can be obtained by aggregating the component scores. The overall score range from 0 to 21 with a higher score indicating a poorer sleep quality. The validity and reliability of CPSQI have been confirmed in Chinese populations.[35-37] The CPSQI has a

Cronbach's α coefficient 0.79 and a good test-retest reliability (r = 0.79, *P* = 0.01) for a 3-week interval.[3]

Health-related quality of life (HRQoL)

The Chinese Severe Respiratory Insufficiency questionnaire (CSRI) will be used to assess the participants' HRQoL.[38] The CSRI consists a total of 49 items for seven subscales (i.e., respiratory complaints, physical functioning, attendant symptoms and sleep, social relationships, anxiety, psychological well-being, and social functioning). Each item is rated on a 5-point Likert scale ranging from 1=completely untrue to 5=always true. Subscale scores can be summed up to obtain a total score. After transformation of raw scores, subscale and total scale scores range 0-100. It has been validated in Chinese COPD patients with hypercapnia and the reported Cronbach's α coefficients were all above 0.7.[38]

Venous bicarbonate (HCO_3^-)

The subject's venous blood sample of 3-5 ml will be collected for laboratory tests of venous HCO_3^- which is considered as less invasive and a reliable estimation for level of hypercapnia.[39-40] The normal range for venous HCO_3^- is 23-29 mmol/L.

Hospital admissions and survival rate

The subject's number of emergence room and unplanned hospital admissions related to hypercapnic respiratory failure at 7th week, 3-, 6-, and 12 months after baseline and survival information at 7th week, 3-, 6-, and 12 months after baseline will be obtained from the subject's medical record via the Clinical Management System (CMS) of Hospital Authority.

6. Data collection procedure

Patients will be recruited by the RA from the respiratory clinics, according to subject inclusion and exclusion criteria. The RA will explain to eligible patients the purpose of the study, their commitment and their right to withdraw at any time. Formal written consent will be obtained from patients. Then the RA will collect the participant's socio-demographic and clinical characteristics, and baseline data (i.e., domiciliary NIV adherence in the past two weeks, CPSQI, CSRI, and number of unplanned hospital admissions related to hypercapnic respiratory failure in

the past 6 months). While a doctor of the respiratory team will prescribe collection of venous HCO_3^- for the hospital phlebotomist to collect venous blood sample, the RA will collect the participant's result of venous HCO_3^- from the CMS of the hospital. After collection of baseline data, participants will be randomly assigned to intervention or control group.

The RA will contact the participant by phone to arrange collection of post-interventional data when the time is approaching. The RA will collect post-interventional data (i.e., domiciliary NIV adherence in the past two weeks, CPSQI, and CSRI) at the patient's home according to the study timeline. A doctor of the respiratory team will prescribe collection of venous HCO_3^- again for the hospital phlebotomist to collect venous blood sample with the patient in hospital at 3 months and 6 months after baseline. The RA will retrieve the patient's venous HCO_3^- result accordingly; number of unplanned hospital admissions related to hypercapnic respiratory failure at 7th week, 3, 6, and 12 months after baseline;, and survival information from the CMS of the hospital at 7th week, 3, 6, and 12 months after baseline.

7. Data analysis

The IBM SPSS 29.0 (IBM Crop., Armonk, NY) will be used for data analysis. Data coding will be carried out before data entry. The data will be double-entered for validation and analyzed on an intention-to-treat basis. Data cleansing will be carried out to eliminate data entry errors. Normality of continuous data will be assessed on the basis of skewness and kurtosis statistics and normal probability plot. Suitable transformation will be made on skewed variables if needed. Descriptive statistics including frequency, percentage, mean, median, range, standard deviation and inter-quartile range will be used to summarize socio-demographic and clinical characteristics of the participants and their health outcome measurements (i.e., adherence data, the venous HCO_3^-, CPSQI, CSRI, number of unplanned hospital admissions, and survival rates). Homogeneity of socio-demographic and clinical data between the intervention and control groups will be assessed by using chi-square, Fisher's exact or independent t tests, as appropriate, with adjustment for potential confounders (i.e. baseline group difference in clinical-demographic characteristics at $p<0.25$ (two-sided).

Generalized estimating equations (GEE) models will be used to compare the primary (NIV adherence) and secondary outcomes (the venous HCO_3^-, CPSQI, CSRI) across time points between the two groups. GEE model can account for intra-correlated repeated measures data and

be applicable for various kinds of outcome variables with the use of suitably chosen link function.[9/41] Furthermore, such model can produce unbiased estimates even in the presence of missing data, provided the data are missing at completely random.[41] If the data is not missing at random as indicated by a statistical significant results of the Little's Missing Completely at Random test (MCAR), multiple imputation method will be used to impute missing data, using all available baseline characteristics as covariable. Predictive mean matching approach which relies less on the parametric assumptions of the imputation models will be used.[42] A dummy variable (group) will be assigned to represent the intervention group with the control group as reference. Other dummy variables (Time) representing time differences, the first post-test endpoint (T1), second post-test endpoint (T2 at the 6th month) and the third post-test endpoint (T3 at the 12th month) will be set to correspond to T1, T2 and T3 respectively, with the baseline T0 as the reference. The interaction terms of the time-point dummy variables and group (group*T1, group*T2 and group*T3) will be included in the GEE model to assess the differential changes of each outcome at T1, T2, and/or T3 relative to T0 between the two groups. Adjusted mean difference and the 95% confidence interval of the differential change over time between the two study groups were reported (with the baseline as the reference) and the Cohen d was computed to indicate the effect size of the between group difference in the change from the baseline. Negative binomial regression identified the between-group differences in ER attendance and the hospital admissions because these data may be over-dispersed in the sample. Cox proportional hazards regression analysis was performed to compare the between-group difference in the time to event (i.e. unplanned time to hospital readmission and mortality). All statistical tests involved will be 2-sided with level of significance set at 0.05.

IV. EHICAL CONSIDERATIONS

Ethical approvals for the study from Research Ethics Committee from the Hospital Authority (Kowloon Central/Kowloon East/ Hong Kong West region) before the commencement of the study. The participation is voluntary and written informed consent will be obtained from the participants. All the data collection is anonymous and the confidentiality will be maintained. Only the research team will have access to the data.

V. REFERENCES

1. Shah NM, Steier J, Hart N, *et al.* Effects of non-invasive ventilation on sleep in chronic hypercapnic respiratory failure. Thorax, 2024,**79:**281-288.
2. Chung Y, Garden FL, Marks GB, Vedam H. Population prevalence of hypercapnic respiratory failure from any cause. Am J Respiratory and Critical Care Medicine, 2022: 205(8): 968-969.
3. Meservey AJ, Burton MC, Priest J, Teneback CC, Dixon AE. Risk of Readmission and Mortality Following Hospitalization with Hypercapnic Respiratory Failure. Lung, 2020,198(1):121-134.
4. Raveling T, Vonk J, Struik FM, et al.. Chronic non-invasive ventilation for chronic obstructive pulmonary disease. Cochrane Database System Review, 2021, 8:CD002878. 10.1002/14651858.CD002878.
5. Meecham Jones DJ, Paul EA, Jones PW, Wedzicha JA. Nasal pressure support ventilation plus oxygen compared with oxygen therapy alone in hypercapnic COPD. *Am J Respir Crit Care Med* 1995; 152: 538-44.
6. Elliott MW, Mulvey DA, Moxham J, Green M, Branthwaite MA. Domiciliary nocturnal nasal intermittent positive pressure ventilation in COPD: mechanisms underlying changes in arterial blood gas tensions. *Eur Respir J* 1991; 4: 1044-52.
7. Ambrosino N, Montagna T, Nava S, Negri A, Brega S, Fracchia C, et alZocchi L, Rampulla C. Short term effect of intermittent negative pressure ventilation in COPD patients with respiratory failure. *Eur Respir J* 1990; 3: 502-8.
8. Duiverman ML, Wempe JB, Bladder G, Vonk JM, Zijlstra JG, Kerstjens HA, Wijkstra PJ. Two-year home-based nocturnal noninvasive ventilation added to rehabilitation in chronic obstructive pulmonary disease patients: a randomized controlled trial. *Respir Res* 2011; 12: 112.
9. Suraj KP, Jyothi E, Rakhi R. Role of domiciliary noninvasive ventilation in chronic obstructive pulmonary disease patients requiring repeated admissions with acute type II respiratory failure: A prospective cohort study. *Indian J Crit Care Med* 2018; 22(6). doi: 10.4103/ijccm.IJCCM_61_18

10. Blankenburg T, Benthin C, Pohl S, Bramer A, Kalbitz F, Lautenschlager C, Schutte W. Survival of hypercapnic patients with COPD and obesity hypoventilation syndrome treated with high intensity non-invasive ventilation in the daily routine care. *The Open Respir Med J* 2017; 11: 31-40.
11. Duiverman ML, Windisch W, Storre JH, Wijkstra PJ. The role of NIV in chronic hypercapnic COPD following an acute exacerbation: the importance of patient selection? *Ther Adv Respir Dis* 2016; 10(2): 149-57. doi:10.1177/1753465815624645
12. Kohnlein T, Windisch W, Kohler D, Drabik A, Geiseler J, Hartl S, Karg O, Laier-Groneveld G, Nava S, Schonhofer B, Schucher B, Wegscheider K, Criee CP, Welte T. Noninvasive positive pressure ventilation for the treatment of severe stable chronic obstructive pulmonary disease: a prospective, multicentre, randomised, controlled clinical trial. *Lancet Respir Med* 2014; 2: 698–705.
13. Dreher M, Storre JH, Schmoor C, Windisch W. High-intensity versus low-intensity non-invasive ventilation in patients with stable hypercapnic COPD: a randomised crossover trial. *Thorax* 2010; 65: 303-8.
14. McEvoy RD, Pierce RJ, Hillman D, Esterman A, Ellis EE, Catcheside PG, O'Donoghue FJ, Barnes DJ, Grunstein RR. Nocturnal non-invasive nasal ventilation in stable hypercapnic COPD: a randomised controlled trial. *Thorax* 2009; 64: 561-6. doi:10.1136/thx.2008.108274
15. Altintas N. Update: Non-invasive positive pressure ventilation in chronic respiratory failure due to COPD. *COPD* 2016; 13: 110-21.
16. Murphy PB, Rehal S, Arbane G, Bourke S, Calverley PMA, Crook AM, et al. Effect of home noninvasive ventilation with oxygen therapy vs oxygin therapy alone on hospital readmission or death after an acute COPD exacerbation: A randomized clinical trial. *JAMA* 2017, 317(21): 2177-86. doi: 10.1001/jama.2017.4451
17. Schwarz EI, Mackie M, Weston N, Tincknell L, Beghal G, Cheng MCF, et al. Time-to-death in chronic respiratory failure on home mechanical ventilation: a cohort study. Respiratory Medicine, 2020, 162:105877. 7.
18. Patout M, Lhuillier E, Kaltsakas G, Benattia A, Dupuis J, Arbane G, et al. Long-term survival following initiation of home non-invasive ventilation: a European study. Thorax, 2020;75(11):965-973.

19. Mokhlesi B, Tulaimat A, Evans AT, Wang Y, Itani AA, Hassaballa HA, et al. Impact of adherence with positive airway pressure therapy on hypercapnia in obstructive sleep apnea. Journal of Clinical Seep Medicine, 2006, 2 (1):57-62.
20. Masa JF, Corral J, Alonso ML, Ordax E, Troncoso MF, Gonzalez M, et al. Efficacy of different treatment alternatives for obesity hypoventilation syndrome. Pickwick Study. American Jornal of Respiratory and Critical Care Medicine, 2015, 192(1):86-95.
21. Cheng S, Chan VL, Chu CM. Compliance with home non-invasive ventilation. *Respirology* 2012; 17: 735-6.
22. Beckert, L., Wiseman, R., Pitama, S., and Landers, A. What can we learn from patients to improve their non-invasive ventilation experience? 'It was unpleasant; if I was offered it again, I would do what I was told.'. *BMJ Support. Palliative Care, 2020,* 10:e7. doi: 10.1136/bmjspcare-2016-001151
23. Volpato E, Banfi PI and Pagnini F. Acceptance and adherence to non-invasive positive pressure ventilation in people with chronic obstructive pulmonary disease: a grounded theory study. *Frontier. Psychology,* 2023, 14:1134718.
24. Gale NK, Jawad M, Dave C, Turner AM. Adapting to domiciliary non-invasive ventilation in chronic obstructive pulmonary disease: A qualitative interview study. *Palliative Medicine* 2014; Dec: 1-10. doi: 10.1177/0269216314558327
25. Ennis J, Rohde K, Chaput J, Buchholz A, Katz SL. Facilitators and Barriers to Noninvasive Ventilation Adherence in Youth with Nocturnal Hypoventilation Secondary to Obesity or Neuromuscular Disease. *J Clini Sleep Med* 2015; 11(12): 1409 –16.
26. Fisher WA, Fisher JD, Harman J. The Information-Motivation-Behavioral Skills Model: A general social psychological approach to understanding and promoting health behavior. In Suls, Wallston (Eds.), *Social psychological foundations of health and illness,* 82-106. Cornwall, UK: Blackwell Publishing Ltd, 2003.
27. Chang SJ, Choi S, Kim S, Song M. Intervention strategies based on Information-Motivation-Behavioral Skills Model for health behavior change: a systematic review. *Asian Nurs Res* 2014; 8: 172-81.
28. Mayberry LS, Osborn CY. Empirical validation of the Information-Motivation-Behavioral Skills Model of diabetes medication adherence: A framework for intervention. *Diabetes Care* 2014; doi:10.2337/dc13-1828.

29. Cooperman NA, Richter KP, Bernstein SL, Steinberg ML, Williams JM. Determining smoking cessation related information, motivation, and behavioral skills among opiate dependent smokers in methadone treatment. *Substance Use and Misuse* 2015; 50: 566-581.
30. Kim YI, Park JS. Development and Evaluation of a Joint Health Self-management Program for the Elderly with Knee Osteoarthritis in Communities: Applying the IMB Model. *J Korean Acad Community Health Nurs* 2017; 28(1): 55-68.
31. Nelson LA, Wallston KA, Kripalani S, LeStourgeon LM, Williamson SE, Mayberry LS. Assessing barriers to diabetes medication adherence using the Information-Motivation-Behavioral Skills Model. *Diabetes Res Clini Pract* 2018; 142: 374-84.
32. To KW, Lee FKI. The effect of a theory-driven educational-intervention for improving adherence to inhalation therapy in patients with chronic obstructive pulmonary disease: A pilot study. Presented in *Hong Kong College of Gerontology Nursing Scientific Meeting cum AGM 2018*. Hong Kong: Hong Kong College of Gerontology Nursing, 26 May 2018.
33. Blankenburg T, Benthin C, Pohl S, Bramer A, Kalbitz F, Lautenschlager C, Schutte W. Survival of hypercapnic patients with COPD and obesity hypoventilation syndrome treated with high intensity non-invasive ventilation in the daily routine care. *The Open Respir Med J* 2017; 11: 31-40.
34. Tsai P, Wang S, Wang M, Su C, Yang T, Huang C, Fang S. Psychometric evaluation of the Chinese version of the Pittsburgh Sleep Quality Index (CPSQI) in primary insomnia and control subjects. *Quality of Life* 2005; 14(8): 1943-52.
35. Ho RTH, Fong TCT. Factor structure of the Chinese version of the Pittsburgh Sleep Quality Index in breast cancer patients. *Sleep Med* 2014; 15: 565-9.
36. Cheung LM, Wong WS. The effects of insomnia and internet addiction on depression in Hong Kong adolescents: an exploratory cross-sectional analysis. *J Sleep Res* 2010; 20(2). https://doi.org/10.1111/j.1365-2869.2010.00883.x
37. Wong WS, Fielding R. Prevalence of insomnia among Chinese adults in Hong Kong: a population-based study. *J Sleep Res* 2011; 20(1). https://doi.org/10.1111/j.1365-2869.2010.00822.x

38. Chen R, Guan L, Wu W, Yang Z, Li X, Lui Q, et al. The Chinese version of the Severe Respiratory Insufficiency questionnaire for patients with chronic hypercapnic chronic obstructive pulmonary disease receiving non-invasive positive pressure ventilation. *BMJ Open* 2017; 7:e017712. doi:10.1136/bmjopen-2017-017712
39. Kee JL. *Laboratory and diagnostic tests with nursing implications*, 6th ed.. Upper Saddle River, New Jersey: Prentice Hall, 2002.
40. Malatesha G, Singh NK, Bharija A, Rehani B, Goel A. Comparison of arterial and venous pH, bicarbonate, Pco2 and Po2 in initial emergency department assessment. *Emerg Med J* 2007; 24(8): 569–71. doi: 10.1136/emj.2007.046979
41. Singer JD, Willett JB. Applied longitudinal data analysis: modelling change and event occurrence. New York: Oxford University Press; 2003.
42. Little RJA: Missing-data adjustments in large surveys. Journal of Business & Economic Statistics 1988; 6: 287-96.
