# Supplementary material for: Health Communication and Adherence to Noninvasive Ventilation in Chronic Hypercapnic Respiratory Failure: A Randomized Clinical Trial
Source: JAMA Netw Open. 2024 Dec 26;7(12):e2451614. doi: 10.1001/jamanetworkopen.2024.51614 (PMC11672160; doi:10.1001/jamanetworkopen.2024.51614)
Supplement: Supplement 2. — eTable 1. Statistics on Goal-Setting for the Intervention Group eTable 2. Effects of the Information-Motivation-Behavioral–Noninvasive Ventilation (IMB-NIV) Program on Sleep Quality and Health-Related Quality of Life eTable 3. Subgroup Analysis of the Effects of the Information-Motivation-Behavioral–Noninvasive Ventilation (IMB-NIV) Program on NIV Adherence eTable 4. Subgroup Analysis of the Effects of the Information-Motivation-Behavioral–Noninvasive Ventilation (IMB-NIV) Program on Time to Hospital Service Use eTable 5. Subgroup Analysis of the Effects of the Information-Motivation-Behavioral–Noninvasive Ventilation (IMB-NIV) Program on Number of Emergency Department Admissions and Hospital Admissions [file jamanetwopen-e2451614-s002.pdf]

Supplemental Online Content

Yu DF, Li PWC, Lau JCC, et al. Health communication and adherence to noninvasive ventilation in chronic hypercapnic respiratory failure: a randomized clinical trial. *JAMA Netw Open.* 2024;7(12):e2451614. doi:10.1001/jamanetworkopen.2024.51614

eTable 1. Statistics on Goal-Setting for the Intervention Group

eTable 2. Effects of the Information-Motivation-Behavioral–Noninvasive Ventilation (IMB-NIV) Program on Sleep Quality and Health-Related Quality of Life

eTable 3. Subgroup Analysis of the Effects of the Information-Motivation-Behavioral–Noninvasive Ventilation (IMB-NIV) Program on NIV Adherence

eTable 4. Subgroup Analysis of the Effects of the Information-Motivation-Behavioral–Noninvasive Ventilation (IMB-NIV) Program on Time to Hospital Service Use

eTable 5. Subgroup Analysis of the Effects of the Information-Motivation-Behavioral–Noninvasive Ventilation (IMB-NIV) Program on Number of Emergency Department Admissions and Hospital Admissions

This supplemental material has been provided by the authors to give readers additional information about their work.

© 2024 Yu DF et al. *JAMA Network Open.*

eTable 1. Statistics on Goal-Setting for the Intervention Group

Category of the goals set for the intervention arm to increase the adherence to non-invasive ventilation	Number of patients being prescribed with the goal category N (%)
Category 1: Goals relating to improve symptom management of chronic hypercapnic respiratory failure.	56 (90.3%)
Category 2: Goals relating to reduce discomfort relating to the non-invasive ventilation.	57 (91.9%)
Category 3: Goals relating to increase the self-care on the operation of the non-invasive ventilator.	34 (54.8%)
Category 4: Goals relating to reduce the unfavorable emotional response associated with the use of non-invasive ventilation.	29 (46.8%)
Category 5: Goals relating to improving self-care on management chronic hypercapnic respiratory failure.	44 (71.0%)
Category 6: Goals relating to change the perception and misconception on the use of non-invasive ventilation.	55 (88.7%)
Category 7: Goals relating to increase the social capacity to enable the use of non-invasive ventilation (including reduce social unacceptance, effective help-seeking, and positive dependency)	35 (56.5%)

© 2024 Yu DF et al. *JAMA Network Open.*

eTable 2. Effects of the Information-Motivation-Behavioral–Noninvasive Ventilation (IMB-NIV) Program on Sleep Quality and Health-Related Quality of Life

	**Mean (SD)**		**Treatment effect (95% CI)**		**Time x group interaction effect by GEE**	
**Patient-reported outcome**	**IMB-based Intervention**	**Usual care**	**Difference in change (IMB Intervention–usual care)**	**Effect size (Cohen d)**	**B (mea95% CI)**	***P* value**
**PSQI – P1 sleep quality**						
Baseline	1.50 (0.76)	1.50 (0.80)	N/A	N/A	N/A	N/A
T1, posttest 7th week;	1.51 (0.67)	1.68 (0.80)	-0.17 (-0.50 - 0.16)	-.186	-0.17 (-0.50 - 0.15)	.300
T2, posttest 3rd month;	1.26 (0.60)	1.68 (0.72)	-0.42 (-0.72 - -0.11)	-.493	-0.44 (-0.73 - -0.14)	**.004***
T3, posttest 6th month	1.17 (0.69)	1.68 (0.66)	-0.55 (-0.89 - -0.21)	-.594	-0.53 (-0.86 - -0.21)	**.001***
T4, posttest 12th month	0.96 (0.57)	1.60 (0.76)	-0.66 (-0.98 - -0.34)	-.780	-0.63 (-0.94 - -0.33)	**<.001***
**PSQI – P2 sleep latency**						
Baseline	1.56 (0.92)	1.68 (1.01)	N/A	N/A	N/A	N/A
T1, posttest 7th week;	1.43 (0.85)	1.73 (0.96)	-0.32 (-0.72 - 0.08)	-.647	-0.34 (-0.73 - 0.06)	.093
T2, posttest 3rd month;	1.42 (0.87)	1.89 (0.97)	-0.50 (-0.90 - -0.10)	-.813	-0.53 (-0.92 - -0.13)	**.009***
T3, posttest 6th month	1.05 (0.67)	1.64 (0.99)	-0.71 (-1.13 - -0.29)	-.999	-0.72 (-1.13 - -0.32)	**<.001***
T4, posttest 12th month	1.56 (1.02)	1.61 (1.06)	-0.81 (-1.25 - -0.37)	-1.074	-0.83 (-1.25 - -0.42)	**<.001***
**PSQI – P3 sleep duration**						
Baseline	1.56 (1.02)	1.61 (1.06)	N/A	N/A	N/A	N/A
T1, posttest 7th week;	1.80 (0.89)	2.08 (0.75)	-0.16 (-0.58 - 0.26)	-.139	-0.22 (-0.63 - 0.20)	.311
T2, posttest 3rd month;	1.39 (1.02)	1.88 (0.87)	-0.37 (-0.78 - 0.05)	-.320	-0.45 (-0.86 - -0.04)	**.031***
T3, posttest 6th month	1.30 (0.94)	1.93 (0.93)	-0.50 (-0.96 - -0.04)	-.403	-0.60 (-1.04 - -0.15)	**.009***
T4, posttest 12th month	1.21 (0.89)	2.02 (0.76)	-0.68 (-1.13 - -0.24)	-.578	-0.78 (-1.21 - -0.35)	**<.001***
**PSQI – P4 habitual sleep efficiency**						
Baseline	1.03 (1.24)	0.92 (1.22)	N/A	N/A	N/A	N/A
T1, posttest 7th week;	1.48 (1.18)	1.46 (1.19)	-0.12 (-0.67 - 0.44)	-.075	-0.11 (-0.66 - 0.44)	.697
T2, posttest 3rd month;	1.07 (1.06)	1.47 (1.19)	-0.54 (-1.08 - -0.01)	-.369	-0.58 (-1.09 - 0.07)	**.025***
T3, posttest 6th month	1.03 (1.12)	1.52 (1.31)	-0.66 (-1.24 - -0.75)	-.415	-0.62 (-1.19 - -0.04)	**.035***
T4, posttest 12th month	1.02 (1.21)	1.53 (1.20)	-0.73 (-1.28 - -0.18)	-.498	-0.62 (-1.16 - -0.09)	**.023***
**PSQI – P5 sleep disturbance**						
Baseline	1.23 (0.49)	1.23 (0.53)	N/A	N/A	N/A	N/A
T1, posttest 7th week;	1.10 (0.40)	1.12 (0.49)	-0.01 (-0.22 - 0.20)	-.022	-0.03 (-0.24 - 0.17)	.752
T2, posttest 3rd month;	0.98 (0.34)	1.25 (0.54)	-0.26 (-0.49 - -0.04)	-.420	-0.27 (-0.49 - -0.05)	**.017***
T3, posttest 6th month	0.97 (0.32)	1.13 (0.43)	-0.14 (-0.37 - 0.09)	-.228	-0.18 (-0.40 - 0.04)	.113
T4, posttest 12th month	1.04 (0.33)	1.25 (0.48)	-0.23 (-0.47 - 0.01)	-.363	-0.24 (-0.46 - -0.01)	**.040***
**PSQI – P6 use of sleep medication**						
Baseline	0.37 (0.94)	0.37 (0.91)	N/A	N/A	N/A	N/A
T1, posttest 7th week;	0.49 (1.04)	0.36 (0.91)	0.15 (-0.16 - 0.46)	.172	0.14 (-0.17 - 0.45)	.362
T2, posttest 3rd month;	0.51 (1.04)	0.36 (0.91)	0.16 (-0.18 - 0.51)	.175	0.16 (-0.18 - 0.49)	.354
T3, posttest 6th month	0.33 (0.86)	0.61 (1.04)	-0.30 (-0.65 - 0.05)	-.312	-0.30 (-0.64 - 0.05)	.094
T4, posttest 12th month	0.52 (0.95)	0.65 (0.99)	-0.45 (-0.55 - 0.25)	-.140	-0.16 (-0.55 - 0.22)	.403
**PSQI – P7 day-time dysfunction**						
Baseline	1.05 (0.98)	0.92 (1.00)	N/A	N/A	N/A	N/A
T1, posttest 7th week;	0.90 (0.75)	1.05 (0.88)	-0.32 to (-0.69 - 0.05)	-.309	-0.30 (-0.66 - 0.07)	.111
T2, posttest 3rd month;	0.87 (0.69)	1.20 (0.87)	-0.50 (-0.94 - -0.07)	-.418	-0.49 (-0.92 - -0.07)	**.023***
T3, posttest 6th month	0.82 (0.68)	1.46 (0.89)	-0.80 (-1.22 - -0.39)	-.715	-0.80 (-1.20 - -0.40)	**<.001***
T4, posttest 12th month	0.71 (0.71)	1.45 (0.86)	-0.89 (-1.35 - -0.42)	-.718	-0.89 (-1.34 - -0.45)	**<.001***
**CSRI – respiratory complaints**						
Baseline	58.82 (21.24)	64.06 (23.74)	N/A	N/A	N/A	N/A
T1, posttest 7th week;	59.22 (16.47)	55.51 (17.54)	8.51 (0.55 - 16.46)	.387	8.68 (0.88 - 16.48)	**.029***
T2, posttest 3rd month;	63.22 (13.98)	54.29 (19.92)	13.72 (5.90 - 21.54)	.635	14.40 (6.78 - 22.03)	**<.001***
T3, posttest 6th month	68.18 (15.26)	56.98 (16.36)	16.64 (7.23 - 26.05)	.651	17.16 (8.11 - 26.21)	**<.001***
T4, posttest 12th month	69.53 (14.09)	56.76 (14.42)	18.05 (0.42 - 26.69)	.787	18.09 (9.94 - 26.25)	**<.001***
**CSRI – physical functioning**						
Baseline	54.37 (14.42)	60.48 (27.17)	N/A	N/A	N/A	N/A
T1, posttest 7th week;	65.30 (20.69)	61.86 (23.96)	9.64 (2.22 - 17.06)	.470	9.58 (2.27 - 16.90)	**.010***
T2, posttest 3rd month;	69.74 (19.18)	62.29 (24.66)	13.66 (5.37 - 21.95)	.596	13.43 (5.30 - 21.56)	**.001***

© 2024 Yu DF et al. *JAMA Network Open.*

T3, posttest 6th month	69.03 (19.46)	62.50 (22.77)	12.70 (4.45 - 20.95)	.567	12.87 (4.95 - 20.78)	**.001***
T4, posttest 12th month	69.64 (18.86)	66.21 (22.66)	9.73 (1.22 - 18.24)	.430	9.49 (1.44 - 17.53)	**.021***
**CSRI – attendant symptoms and sleep**						
Baseline	58.12 (17.90)	58.41 (21.31)	N/A	N/A	N/A	N/A
T1, posttest 7th week;	59.07 (18.88)	50.85 (19.02)	8.14 (0.01 - 16.27)	.362	8.18 (0.19 - 16.18)	**.045***
T2, posttest 3rd month;	63.41 (15.60)	51.09 (18.89)	12.23 (3.92 - 20.55)	.532	12.36 (4.23 - 20.48)	**.003***
T3, posttest 6th month	64.23 (16.69)	52.68 (17.11)	11.51 (2.64 - 20.39)	.478	12.59 (4.13 - 21.05)	**.004***
T4, posttest 12th month	71.56 (13.48)	53.57 (18.85)	17.31 (8.32 - 26.31)	.724	18.71 (10.22 - 27.20)	**<.001***
**CSRI – social relationship**						
Baseline	73.25 (15.93)	73.59 (17.48)	N/A	N/A	N/A	N/A
T1, posttest 7th week;	73.16 (16.61)	72.39 (16.36)	0.49 (-6.13 - 7.12)	.027	0.65 (-5.84 - 7.13)	.846
T2, posttest 3rd month;	76.57 (14.99)	70.48 (19.41)	5.82 (-1.11 - 12.74)	.304	6.44 (-0.37 - 13.25)	.064
T3, posttest 6th month	77.78 (13.19)	70.68 (16.97)	6.66 (0.73 - 12.58)	.414	7.40 (1.69 - 13.10)	**.011***
T4, posttest 12th month	81.25 (11.54)	74.09 (17.06)	6.91 (0.50 - 13.31)	.406	7.62 (1.44 - 13.81)	**.016***
**CSRI – anxiety**						
Baseline	69.27 (20.80)	76.37 (23.51)	N/A	N/A	N/A	N/A
T1, posttest 7th week;	62.21 (23.28)	58.05 (27.32)	10.66 (1.50 - 19.82)	.421	11.14 (2.10 - 20.18)	**.016***
T2, posttest 3rd month;	68.85 (20.03)	55.59 (26.26)	19.76 (11.33 - 28.19)	.848	20.26 (11.94 - 28.57)	**<.001***
T3, posttest 6th month	71.08 (23.83)	60.89 (26.22)	17.99 (6.98 - 29.01)	.601	18.10 (7.36 - 28.85)	**<.001***
T4, posttest 12th month	81.61 (15.99)	64.72 (21.95)	24.51 (14.77 - 34.26)	.947	23.94 (14.38 - 33.49)	**<.001***
**CSRI – psychological wellbeing**						
Baseline	66.44 (19.80)	68.95 (21.10)	N/A	N/A	N/A	N/A
T1, posttest 7th week;	73.73 (15.63)	68.08 (17.92)	7.38 (0.12 - 14.65)	.367	7.55 (0.41 - 14.70)	**.038***
T2, posttest 3rd month;	77.78 (15.15)	65.63 (19.42)	13.89 (6.67 - 21.10)	.696	14.53 (7.45 - 21.60)	**<.001***
T3, posttest 6th month	76.20 (15.46)	69.14 (16.58)	9.28 (1.28 - 17.27)	.427	9.93 (2.26 - 17.60)	**.011***
T4, posttest 12th month	80.36 (13.27)	69.24 (17.18)	12.70 (5.29 - 20.12)	.644	13.65 (6.44 - 20.86)	**<.001***
**CSRI – social functioning**						
Baseline	60.53 (22.21)	65.88 (22.24)	N/A	N/A	N/A	N/A
T1, posttest 7th week;	62.70 (18.33)	59.48 (23.62)	8.44 (1.08 - 15.81)	.415	8.33 (1.08 - 15.58)	**.024***
T2, posttest 3rd month;	67.62 (18.43)	57.31 (23.68)	15.53 (8.21 - 22.85)	.767	15.54 (8.36 - 22.71)	**<.001***
T3, posttest 6th month	66.09 (18.43)	60.32 (20.08)	11.70 (4.04 - 19.36)	.562	11.80 (4.47 - 19.12)	**.002***
T4, posttest 12th month	72.21 (17.75)	65.17 (19.49)	13.09 (5.34 - 20.84)	.636	12.77 (5.52 - 20.02)	**<.001***

Pittsburg Sleep Quality Index (PSQI): range of score = 0-21, with higher score indicating poorer sleep quality; PSQI subscale score = 1-7, with higher score indicating poorer sleeping function; Chinese Severe Respiratory Insufficiency Questionnaire (CSRI): range of score = 0-100, with higher score indicating better health-related quality of life.

© 2024 Yu DF et al. *JAMA Network Open.*

eTable 3. Subgroup Analysis of the Effects of the Information-Motivation-Behavioral–Noninvasive Ventilation (IMB-NIV) Program on NIV Adherence

Patient-reported outcome	Mean (SD) IMB-based Intervention	Usual care	Treatment effect (95% CI) Difference in change (IMB Intervention–usual care)	Effect size (Cohen d)	Time x group interaction effect by GEE B (95% CI)	*P* value
**Subjects with chronic obstructive pulmonary disease as the etiology of chronic hypercapnic respiratory failure (N: intervention vs control = 17 vs 14)**						
**NIV use >4hour days (%)**						
Baseline	54.19 (33.50)	39.21 (32.41)	N/A	N/A	N/A	N/A
T1, posttest 7th week;	78.01 (32.73)	29.61 (38.53)	32.04 (1.15 to 62.93)	.892	32.60 (5.39 to 59.81)	**.019***
T2, posttest 3rd month;	74.95 (36.04)	24.66 (41.90)	33.94 (2.01 to 65.88)	.896	34.89 (6.70 to 63.09)	**.015***
T3, posttest 6th month	66.80 (45.10)	31.83 (40.85)	22.39 (-13.93 to 58.72)	.527	20.67 (-10.22 to 51.55)	.190
T4, posttest 12th month	72.52 (37.03)	35.87 (39.22)	20.99 (-10.88 to 52.85)	.566	23.54 (-3.93 to 51.02)	.093
**NIV mean hour usage daily**						
Baseline	3.76 (1.74)	2.57 (1.86)	N/A	N/A	N/A	N/A
T1, posttest 7th week;	5.92 (2.32)	2.28 (2.42)	2.23 (0.78 to 3.68)	1.158	2.28 (0.86 to 3.70)	**.002***
T2, posttest 3rd month;	5.88 (3.09)	2.23 (2.55)	2.23 (0.32 to 4.15)	.879	2.32 (0.58 to 4.06)	**.009***
T3, posttest 6th month	5.70 (3.79)	2.07 (2.47)	2.55 (0.08 to 5.02)	.811	2.35 (0.17 to 4.52)	**.034***
T4, posttest 12th month	6.15 (3.41)	2.56 (2.90)	2.31 (-0.09 to 4.70)	.768	2.56 (0.46 to 4.66)	**.017***
**NIV use>4 hour days for >70% of the days (no. of participants (%))^**						
Baseline	0 (0.0)	0 (0.0)	N/A			N/A
T1, posttest 7th week;	11 (64.71)	1 (7.14)	0.05 (0.01 to 0.44)			**.002***
T2, posttest 3rd month;	11 (64.71)	3 (21.43)	0.16 (0.03 to 0.83)			**.024***
T3, posttest 6th month	11 (64.71)	2 (14.29)	0.09 (0.01 to 0.58)			**.006***
T4, posttest 12th month	9 (52.94)	3 (21.43)	0.22 (0.04 to 1.18)			.069
**Subjects with obstructive sleep apnea comorbid with other chronic respiratory disorder as the etiological factors of chronic hypercapnic respiratory failure (N: intervention vs control = 21 vs 24)**						
**NIV use >4hour days (%)**						
Baseline	45.51 (32.31)	38.81 (32.03)	N/A	N/A	N/A	N/A
T1, posttest 7th week;	68.43 (40.94)	41.50 (34.35)	15.64 (0.09 to 31.20)	.628	16.42 (1.90 to 30.94)	**.027***
T2, posttest 3rd month;	64.07 (40.79)	49.50 (42.11)	5.01 (-13.29 to 23.30)	.173	6.69 (-10.15 to 23.53)	.436
T3, posttest 6th month	67.11 (45.55)	42.49 (37.25)	17.44 (-0.92 to 35.79)	.602	17.96 (0.47 to 35.45)	**.044***
T4, posttest 12th month	60.12 (44.90)	45.01 (44.90)	5.72 (-14.28 to 25.73)	.185	5.39 (-13.16 to 23.94)	.569
**NIV mean hour usage daily**						
Baseline	3.39 (1.66)	2.89 (1.86)	N/A	N/A	N/A	N/A
T1, posttest 7th week;	5.38 (2.75)	2.84 (2.16)	1.76 (0.63 to 2.89)	.973	1.80 (0.73 to 2.87)	**<.001***
T2, posttest 3rd month;	5.05 (3.23)	3.23 (2.72)	1.17 (-0.32 to 2.66)	.498	1.26 (-0.14 to 2.65)	.078
T3, posttest 6th month	5.52 (3.17)	2.96 (2.64)	2.02 (0.55 to 3.49)	.869	1.96 (0.52 to 3.40)	**.008***
T4, posttest 12th month	4.61 (3.35)	2.93 (2.36)	0.91 (-0.52 to 2.34)	.414	1.11 (-0.37 to 2.59)	.142
**NIV use>4 hour days for >70% of the days (no. of participants (%))^**						
Baseline	0 (0.0)	0 (0.0)	N/A			N/A
T1, posttest 7th week;	12 (60.0)	2 (9.1)	0.067 (0.012 to 0.367)			**<.001***

© 2024 Yu DF et al. *JAMA Network Open.*

T2, posttest 3rd month;	11 (55.0)	6 (28.6)	0.327 (0.090 to 1.193)			.086
T3, posttest 6th month	13 (68.4)	5 (22.7)	0.136 (0.034 to 0.545)			**.003***
T4, posttest 12th month	9 (52.9)	4 (17.4)	0.187 (0.044 to 0.789)			**.018***
**Subjects with obstructive sleep apnea comorbid as the etiology of chronic hypercapnic respiratory failure (N: intervention vs control = 9 vs 10)**						
**NIV use >4hour days (%)**						
Baseline	43.51 (34.08)	46.62 (35.75)	N/A	N/A	N/A	N/A
T1, posttest 7th week;	76.91 (30.86)	29.32 (28.87)	50.70 (24.97-76.44)	1.910	50.70 (27.84-73.57)	**<.001***
T2, posttest 3rd month;	79.26 (29.14)	21.09 (31.13)	61.28 (29.32-93.24)	1.859	61.28 (14.14-33.56)	**<.001***
T3, posttest 6th month	73.01 (32.31)	23.97 (34.47)	57.33 (24.85-89.82)	1.764	54.21 (14.03-26.71)	**<.001***
T4, posttest 12th month	83.04(34.98)	26.79 (28.26)	63.79 (26.89-100.69)	1.854	63.81 (33.33-94.28)	**<.001***
**NIV mean hour usage daily**						
Baseline	3.06 (2.05)	2.91 (1.95)	N/A	N/A	N/A	N/A
T1, posttest 7th week;	5.85 (2.31)	1.97 (1.82)	3.72 (1.95- 5.50)	2.031	3.72 (2.13- 5.32)	**<.001***
T2, posttest 3rd month;	6.18 (2.02)	1.63 (1.95)	4.36 (2.56- 6.17)	2.340	4.40 (2.81-5.99)	**<.001***
T3, posttest 6th month	5.86 (2.75)	1.91 (2.02)	4.12 (1.72 - 6.52)	1.716	3.96 (1.89-6.04)	**<.001***
T4, posttest 12th month	6.51 (2.97)	1.83 (1.61)	4.80 (2.13- 7.46)	1.932	4.90 (2.75-7.05)	**<.001***
**NIV use>4 hour days for >70% of the days (no. of participants (%))^**						
Baseline	0 (0.0)	0 (0.0)	N/A			N/A
T1, posttest 7th week;	6 (66.67)	1 (10.0)	0.056 (0.005 to 0.669)			**.011***
T2, posttest 3rd month;	8 (88.89)	1 (10.0)	0.014 (0.001 to 0.260)			**<.001***
T3, posttest 6th month	6 (66.67)	1 (10.0)	0.063 (0.005 to 0.760)			**.016***
T4, posttest 12th month	7 (77.78)	0 (0.0)	N/A			**<.001***

© 2024 Yu DF et al. *JAMA Network Open.*

eTable 4. Subgroup Analysis of the Effects of the Information-Motivation-Behavioral–Noninvasive Ventilation (IMB-NIV) Program on Time to Hospital Service Use

	Emergence room admission<br>Hazard ratio (95% confidence interval)[a]	Hospital admission<br>Hazard ratio (95% confidence interval)[a]
CHRF subgroup with COPD as the relating etiology,<br>N (IMB-NIV vs usual care) = 17vs14	0.393 (0.140 – 1.103)	0.457 (0.165 – 1.265)
CHRF subgroup with OSA comorbid with other chronic respiratory disease as the relating etiology,<br>N (IMB-NIV vs usual care) = 21vs24	0.534 (0.145 – 1.968)	0.534 (0.145 – 0.168)
CHRF subgroup wit OSA[d] as the relating etiology,<br>N (IMB-NIV vs usual care) = 9vs10	2.0068 (0.089 – 48.099)	820.158 (0 – 13705063023)

CHRF = Chronic hypercapnic respiratory failure; COPD = chronic obstructive pulmonary disease; OSA = Obstructive sleep apnea; [a] The Cox proportional hazards regression model has adjusted for the age, Charlson Comorbidity Index, and smoking history.

© 2024 Yu DF et al. *JAMA Network Open.*

eTable 5. Subgroup Analysis of the Effects of the Information-Motivation-Behavioral–Noninvasive Ventilation (IMB-NIV) Program on Number of Emergency Department Admissions and Hospital Admissions

	Emergence room admission[a]			Hospital admission[a]		
	Total number of admissions		Incident risk ratios (95% confidence interval)	Total number of admissions		Incident risk ratios (95% confidence interval)
	IMB-NIV	usual care		IMB-NIV	usual care	
CHRF subgroup with COPD[b] as the relating etiology, N (IMB-NIV vs usual care) = 17vs14	13	44	0.234 (0.088 – 0.623)	20	41	0.399 (0.160 – 0.994)
CHRF subgroup with OSA comorbid with other chronic respiratory disease[c] as the relating etiology, N (IMB-NIV vs usual care) = 21vs24	24	7	1.056 (0.276 – 4.042)	16	7	2.508 (0.770-8.172)
CHRF subgroup wit OSA[d] as the relating etiology N (IMB-NIV vs usual care) = 9vs10	1	1	1.667 (0.069 – 38.643)	1	1	0.00

CHRF = Chronic hypercapnic respiratory failure; COPD = chronic obstructive pulmonary disease; OSA = Obstructive sleep apnea; [a] The binominal negative regression model has adjusted for the age, Charlson Comorbidity Index, and smoking history.

© 2024 Yu DF et al. *JAMA Network Open.*
