# Supplementary material for: Health Communication and Adherence to Noninvasive Ventilation in Chronic Hypercapnic Respiratory Failure: A Randomized Clinical Trial
Source: JAMA Netw Open. 2024 Dec 26;7(12):e2451614. doi: 10.1001/jamanetworkopen.2024.51614 (PMC11672160; doi:10.1001/jamanetworkopen.2024.51614)
Supplement: Supplement 3. — Data Sharing Statement [file jamanetwopen-e2451614-s003.pdf]

Data Sharing Statement

Yu. Health Communication and Adherence to Noninvasive Ventilation in Chronic Hypercapnic Respiratory Failure. *JAMA Netw Open*. Published December 18, 2024.
doi:10.1001/jamanetworkopen.2024.51614

Data
Additional Information: ClinicalTrials.gov; NCT05008211
https://clinicaltrials.gov/study/NCT05008211?cond=chronic%20hypercapnic%20respiratory%20failure&intr=Information-Motivation-Behavioral%20Skills%20Model&rank=1
Data available: No

Additional Information
Explanation for why data not available: To comply with the condition set by the Clinical Ethics Committee that only the research team can have access to the data. Referring to the patient consent, data sharing has not been mentioned.
